# Association between total bilirubin and gender-specific incidence of fundus arteriosclerosis in a Chinese population: a retrospective cohort study

**DOI:** 10.1038/s41598-023-38378-1

**Published:** 2023-07-11

**Authors:** Yongfei Dong, Chunxing Liu, Jieli Wang, Huijun Li, Qi Wang, Aicheng Feng, Zaixiang Tang

**Affiliations:** 1 Department of Biostatistics, School of Public Health, MOE Key Laboratory of Geriatric Diseases and Immunology, Suzhou Medical College of Soochow University, Suzhou, 215123 Jiangsu Province P.R. China; 2 Jiangsu Key Laboratory of Preventive and Translational Medicine for Geriatric Diseases, Suzhou Medical College of Soochow University, Suzhou, 215123 Jiangsu Province China; 3 Department of Laboratory, Hua Dong Sanatorium, Wuxi, 214065 Jiangsu Province China; 4 Department of Otorhinolaryngology, Hua Dong Sanatorium, Wuxi, 214065 Jiangsu Province China

**Keywords:** Retinal diseases, Epidemiology

## Abstract

To investigate the gender-specific relationship between total bilirubin (TBIL) and fundus arteriosclerosis in the general population, and to explore whether there is a dose–response relationship between them. In a retrospective cohort study, 27,477 participants were enrolled from 2006 to 2019. The TBIL was divided into four groups according to the quartile. The Cox proportional hazards model was used to estimate the HRs with 95% CIs of different TBIL level and fundus arteriosclerosis in men and women. The dose–response relationship between TBIL and fundus arteriosclerosis was estimated using restricted cubic splines method. In males, after adjusting for potential confounders, the Q2 to Q4 level of TBIL were significantly associated with the risk of fundus arteriosclerosis. The HRs with 95% CIs were 1.217 (1.095–1.354), 1.255 (1.128–1.396) and 1.396 (1.254–1.555), respectively. For females, TBIL level was not associated with the incidence of fundus arteriosclerosis. In addition, a linear relationship between TBIL and fundus arteriosclerosis in both genders (*P* < 0.0001 and *P* = 0.0047, respectively). In conclusion, the incidence of fundus arteriosclerosis is positively correlated with serum TBIL level in males, but not in females. In addition, there was a linear dose–response relationship between TBIL and incidence of fundus arteriosclerosis.

## Introduction

Atherosclerosis is caused by chronic inflammation of the arterial vasculature and lipid deposition in the vessel wall, which is a progressive age-related process that is the leading cause of death worldwide. The main mechanisms of atherosclerosis formation include chronic inflammation, autoimmune response, and infection^1–4^. Atherosclerosis can cause a decrease in blood flowed to either large or small vessels throughout the body, which in turn leads to ischemic damage to organs^5^. Microatherosclerosis is a small vessel lesion that occurs to the small terminal arteries or capillaries. The human retina provides a unique model for studying small vessel disease that reflects the pathological features of small vessels throughout the body, including the coronary microcirculation^6,7^. Microvascular retinal disease is often seen as a complication in hypertensive and diabetic populations, and is therefore commonly used for diagnosis or stratification^8,9^. In previous studies, retinal microangiopathy was considered a biomarker to predict the development of cerebrovascular ischemic disease, while it is noted that retinal vasculopathy is associated with brain disorders such as cognitive decline, hearing loss, and dementia^8,10–12^. In addition, fundus atherosclerosis was considered a sign of atherosclerosis and was thought to be associated with a variety of cardiovascular diseases (CVD)^13,14^.

Total bilirubin (TBIL) includes direct bilirubin (DBIL) and indirect bilirubin (IBIL). Bilirubin is produced during the catabolism of heme and is processed further in the liver to form water-insoluble IBIL, which is combined with uridine diphosphate glucuronyl transferase to form DBIL, which is excreted by the kidney^15^. Bilirubin plays a key role as a clinical indicator of liver function. For example, elevated DBIL may indicate hepatocyte damage^16^. As an endogenous antioxidant with antioxidant, anti-inflammatory, and anti-lipogenic effects. Bilirubin has been identified, in several recent reviews, as playing a key role in the pathogenesis of cardiovascular events such as atherosclerosis and metabolic diseases^17–19^. The anti-atherosclerosis mechanism of bilirubin mainly includes vascular smooth muscle cell proliferation and endothelial cell dysfunction^18,20^. Previous population-based epidemiological studies have shown that mild hyperbilirubinemia protects against diabetic microangiopathy in people with diabetes^21^. Whereas, other studies have shown that bilirubin plays a dual role in cells, with high bilirubin levels, a cytotoxic metabolite, also causes oxidative stress responses and neurotoxicity that can harm the brain, like Parkinson's disease (PD)^20,22,23^. Although existing epidemiological studies had shown that mildly elevated bilirubin has a protective effect on atherosclerosis, its high concentrations can still cause oxidative stress. Given these inconsistent results, in addition, the relationship between bilirubin and atherosclerosis in small retinal vessels is poorly studied.

Because of this, we conducted a longitudinal study to analyze the relationship between TBIL and the presence of fundus arteriosclerosis in a general medical examination population. Furthermore, we aimed to explore if there is a dose–response relationship between these two variables.

## Materials and methods

### Study population

**Figure 1:**
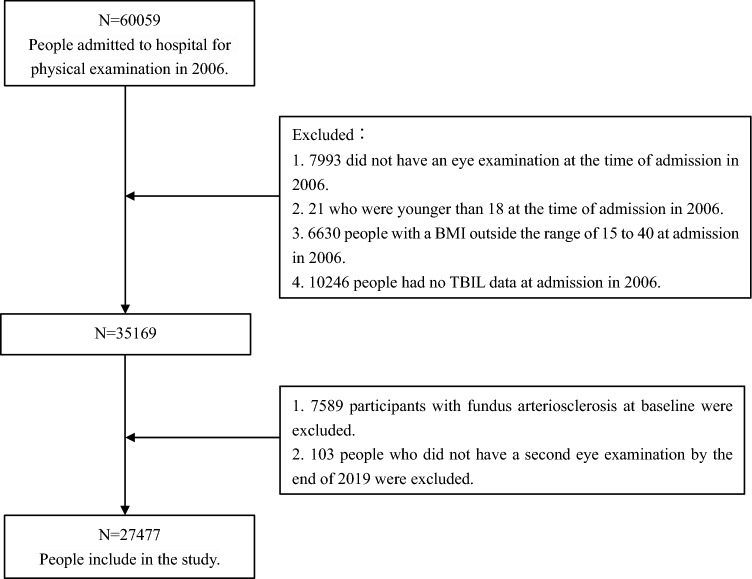
Describe the sample flow chart for screening studies for statistical analysis.

The study population was analyzed from January 2006 to December 2019 in Hua Dong Sanatorium. A total of 60,059 people had a health examination at the time of admission in 2006. 7993 participants lacked an eye exam, 21 had younger than 18 years, 6630 had a BMI outside of 15 to 40, and 10,246 had missing information on total bilirubin. In addition, 7589 participants that already had fundus arteriosclerosis at admission, and 103 that did not have a second fundus examination at the end of follow-up in 2019 were excluded. The data processing steps are shown in Fig. 1. A total of 27,477 participants were included, including 17,736 males (64.5%) and 9741 females (35.5%) in the study. This study was approved by the Ethics committee and institutional Review board of Huadong Sanatorium, Wuxi (No.1, 2021). Informed consent was waived, and the ethics committee of Huadong Sanatorium endorsed the need for this waiver. All methods were performed in accordance with the Declaration of Helsinki and relevant guidelines. The personal information of the study subjects was confidential.

### Demographic and clinical information

A questionnaire was administered to all participants by a standard trained professional to obtain information on demographic characteristics and poor lifestyle. The questionnaire included information on age, gender, and questions related to the diagnosis and treatment of diabetes, hypertension, and fatty liver. Adverse lifestyles mainly included smoking and alcohol consumption. Smoking status was divided into current and non-smoking (including never smoked and quitted). Alcohol consumption was classified as current or non-drinking (including never drinking or ever drinking). Participants removed their shoes and hats and stood straight to measure their height and weight. The body mass index (BMI) was calculated by dividing the weight (Kg) by the square of the height (meters) (kg/m^2^). Hypertension was defined as a systolic blood pressure of ≥ 140 mmHg, diastolic blood pressure of ≥ 90 mmHg, or if the participants had been diagnosed with hypertension by an internist or was receiving antihypertensive medication^24^. Diabetes was defined as a fasting blood glucose level of ≥ 7.0 mmol/L, or if the participants had been diagnosed with diabetes by an internist or was receiving oral hypoglycemic agents or insulin therapy^25^.

### Biochemical index measurements

Venous blood samples were collected from each participant in the morning, ensuring that they remained fasted and abstained from food and drink for at least 8 h prior to blood collection. Total bilirubin (TBIL), alanine aminotransferase (ALT), aspartate aminotransferase (AST), gamma-glutamyl transferase (γ-GT), albumin, globulin, total protein (TP), total cholesterol (CHOL), triglycerides (TG), low-density lipoprotein-cholesterol (LDL-C), high-density lipoprotein-cholesterol (HDL-C), fasting glucose (FBG), uric acid (UA) and blood urea nitrogen (BUN) were measured using an AU5400 BECKMAN COULTER automatic biochemistry analyzer.

### Study outcome

The primary outcome measure was the presence or absence of funduscopic arteriosclerosis. Fundus examination was performed on the participants by a professional ophthalmologist. Fundus photography was performed using a non-dilated fundus camera TRC.NW400TOPCON according to the standard protocol^26^. Subjects was diagnosed according to the Keith-Wagner-Barker classification^27^. Grade 1 was retinal artery spasm or mild sclerosis. Grade 2 was moderate to marked sclerosis of retinal arteries with varying degrees of pathological changes visible to the arterioles. Grade 3 was marked sclerosis of retinal arteries with some visible edema, absorbent cotton spots and retinal hemorrhages. Grade 4 was in addition to grade 3 features, visible edema with the optic disc^28^. In this study, participants with any of the grades’ features were considered to have fundus arteriosclerosis.

### Statistical analyses

All participants were divided into four groups based on quartiles of TBIL level, with the first quartile as the reference group. Baseline characteristics were compared in the total population, male and female in each of the four groups. Basic characteristics of participants were reported as medians (quartile intervals) for continuous variables with non-normal distributions and as numbers (percentages) for categorical variables. The Kruskal–Wallis test was used to compare continuous variables while the chi-square test was used to compare categorical variables. The Linear regression or the Cochran-Armitage (CA) trend test was used for trend analysis. The Cox proportional hazards models were used to measure hazard ratios (HRs) and 95% confidence intervals (95% CIs) for changes in TBIL in the whole population, male and female. Cumulative incidence curves were expressed by Kaplan–Meier method, and differences were tested by Log-rank tests. Dose–response relationships for TBIL were assessed by the Restricted Cubic Spline (RCS) Regression Models with knots placed at the 5th, 35th, 65th, and 95th percentiles and by using the 12.5th percentile as the reference category^29^. All statistical analyses were performed by using the R (4.0.2). P value < 0.05 was considered statistically significant. All statistical tests were two-sided.

## Results

### Basic characteristics

Tables 1 and 2 demonstrated the basic characteristics of the male and female participants, respectively. The prevalence rates of fundus atherosclerosis were 17.3% in men and 9.5% in women. Among males, participants with higher TBIL level had higher rates of alcohol consumption, hypertension, diabetes mellitus, and fundus atherosclerosis, compared with those with lower TBIL level. TBIL level was significantly positively correlated with AST, albumin, and HDL, and negatively correlated with TG and BUN. In women, participants with higher TBIL level had lower age, BMI, TG, BUN, and fatty liver incidence compared to those with lower TBIL level, while AST, albumin and HDL were higher in participants with higher TBIL level. There was no significant trend association between the incidence of fundus atherosclerosis in women.

**Table 1:** Baseline characteristics of participants in males.

Characteristics (Median [IQR])	Total bilirubin (μmol/L)				*P* value	*P* for trend
	Q1: ≤ 10.6 *n* = 4561	Q2:10.6–13.3 *n* = 4404	Q3:13.3–16.6 *n* = 4349	Q4: > 16.6 *n* = 4422		
Age, year	48.00 [40.00, 55.00]	48.00 [40.00, 55.00]	48.00 [41.00, 55.00]	48.00 [40.00, 55.00]	0.023	0.519
BMI, kg/m^2^	24.32 [22.58, 26.23]	24.38 [22.50, 26.17]	24.38 [22.46, 26.26]	24.21 [22.15, 26.09]	< 0.001	0.368
ALT, U/L	23.00 [17.00, 34.00]	24.00 [17.00, 35.00]	24.00 [17.00, 36.00]	24.00 [17.00, 36.00]	0.001	0.200
AST, U/L	21.00 [18.00, 25.00]	21.00 [18.00, 26.00]	22.00 [18.00, 27.00]	22.00 [18.00, 27.00]	< 0.001	< 0.001
γ-GT, U/L	28.00 [19.00, 45.00	29.00 [19.00, 46.00]	28.00 [19.00, 47.00]	28.00 [19.00, 49.00]	0.506	0.156
TP, g/L	74.30 [71.85, 76.99]	74.49 [72.01, 77.12]	74.59 [72.06, 77.22]	74.90 [72.37, 77.56]	< 0.001	0.058
Albumin, g/L	46.71 [45.12, 48.29]	46.98 [45.41, 48.60]	47.15 [45.54, 48.72]	47.46 [45.81, 49.08]	< 0.001	< 0.001
Globulin, g/L	27.34 [25.62, 29.58]	27.24 [25.55, 29.45]	27.30 [25.53, 29.37]	27.19 [25.48, 29.42]	0.029	0.444
TG, mmol/L	1.61 [1.11, 2.41]	1.55 [1.09, 2.23]	1.46 [1.03, 2.16]	1.40 [0.97, 2.07]	< 0.001	< 0.001
CHOL, mmol/L	4.54 [4.05, 5.12]	4.65 [4.13, 5.19]	4.62 [4.11, 5.21]	4.56 [4.08, 5.11]	< 0.001	0.773
HDL, mmol/L	1.12 [0.98, 1.29]	1.18 [1.02, 1.35]	1.20 [1.05, 1.38]	1.25 [1.07, 1.45]	< 0.001	< 0.001
LDL, mmol/L	2.77 [2.32, 3.26]	2.85 [2.41, 3.32]	2.85 [2.38, 3.33]	2.76 [2.32, 3.24]	< 0.001	0.994
FBG, mmol/L	5.31 [5.03, 5.64]	5.30 [5.04, 5.65]	5.32 [5.03, 5.67]	5.32 [5.04, 5.72]	0.047	0.112
UA, μmol/L	372.2[329.7,419.8]	373.4[328.2,420.8]	372.0[326.6,419.2]	370.9[326.7,418.3]	0.279	0.984
BUN, mmol/L	5.20 [4.52, 5.98]	5.10 [4.46, 5.86]	5.05 [4.40, 5.79]	5.00 [4.33, 5.77]	< 0.001	0.028
Smoke, *n* (%)						
Non-smoking	1437 (32.0)	1599 (36.8)	1624 (37.7)	1691 (38.6)	< 0.001	< 0.001
Current	3060 (68.0)	2745 (63.2)	2682 (62.3)	2694 (61.4)		
Drunk, *n* (%)						
Non-drinking	1303 (29.0)	1182 (27.2)	1101 (25.6)	1064 (24.3)	< 0.001	< 0.001
Current	3185 (71.0)	3163 (72.8)	3200 (74.4)	3317 (75.7)		
Hypertension, *n* (%)						
No	3854 (84.5)	3699 (84.0)	3561 (81.9)	3635 (82.2)	0.001	0.002
Yes	707 (15.5)	705 (16.0)	788 (18.1)	787 (17.8)		
Diabetes, *n* (%)						
No	4371 (95.8)	4214 (95.7)	4108 (94.5)	4176 (94.4)	0.001	0.001
Yes	190 (4.2)	190 (4.3)	241 (5.5)	246 (5.6)		
Fatty liver, *n* (%)						
No	2612 (57.3)	2461 (55.9)	2493 (57.3)	2648 (59.9)	0.002	0.014
Yes	1949 (42.7)	1943 (44.1)	1856 (42.7)	1774 (40.1)		
Fundus atherosclerosis, *n* (%)						
No	3894 (85.4)	3620 (82.2)	3541 (81.4)	3605 (81.5)	< 0.001	< 0.001
Yes	667 (14.6)	784 (17.8)	808 (18.6)	817 (18.5)		

ALT, alanine aminotransferase; AST, aspartate aminotransferase; γ-GT, γ-glutamyl transpeptidase; TP, serum total protein; TG, triacylglycerol; CHOL, cholesterol; HDL, high density lipoprotein; LDL, low Density Lipoprotein; FBG, fasting blood glucose; UA, uric acid; BUN, blood urea nitrogen; IQR: interquartile range.

**Table 2:** Baseline characteristics of participants in females.

Characteristics (Median [IQR])	Total Bilirubin, μmol/L				*P* value	*P* for trend
	Q1: ≤ 9.4 *n* = 2518	Q2: 9.4–11.7 *n* = 2395	Q3: 11.7–14.4 *n* = 2406	Q4: > 14.4 *n* = 2422		
Age, year	47.00 [38.00, 54.00]	47.00 [39.00, 54.00]	47.00 [38.00, 54.00]	45.00 [37.00, 52.00]	< 0.001	0.492
BMI, kg/m^2^	22.15 [20.55, 24.13]	21.97 [20.30, 24.01]	21.64 [20.05, 23.73]	21.56 [19.81, 23.53]	< 0.001	0.008
ALT, U/L	14.00 [11.00, 20.00]	15.00 [11.00, 21.00]	15.00 [11.00, 20.00]	15.00 [11.00, 20.00]	0.005	0.331
AST, U/L	18.00 [15.00, 22.00]	18.00 [16.00, 22.00]	19.00 [16.00, 22.00]	18.00 [16.00, 22.00]	< 0.001	0.028
γ-GT, U/L	14.00 [11.00, 19.00]	14.00 [11.00, 20.00]	13.00 [11.00, 19.00]	13.00 [10.00, 18.00]	< 0.001	0.073
TP, g/L	74.56 [71.97, 77.50]	74.82 [72.31, 77.55]	75.00 [72.60, 77.69]	75.13 [72.67, 77.90]	< 0.001	0.293
Albumin, g/L	45.59 [44.07, 47.14]	45.91 [44.37, 47.41]	46.15 [44.61, 47.71]	46.41 [44.93, 48.03]	< 0.001	0.003
Globulin, g/L	28.89 [26.81, 31.00]	28.91 [26.80, 31.03]	28.72 [26.70, 30.96]	28.75 [26.71, 30.70]	0.034	0.358
TG, mmol/L	0.97 [0.70, 1.39]	0.92 [0.67, 1.32]	0.88 [0.65, 1.23]	0.83 [0.62, 1.19]	< 0.001	< 0.001
CHOL, mmol/L	4.46 [3.92, 5.06]	4.58 [4.04, 5.18]	4.54 [4.05, 5.14]	4.60 [4.04, 5.22]	< 0.001	0.712
HDL, mmol/L	1.40 [1.21, 1.63]	1.49 [1.29, 1.70]	1.53 [1.33, 1.74]	1.54 [1.34, 1.77]	< 0.001	< 0.001
LDL, mmol/L	2.59 [2.19, 3.10]	2.66 [2.20, 3.16]	2.60 [2.19, 3.12]	2.64 [2.19, 3.14]	0.170	0.293
FPG, mmol/L	5.19 [4.94, 5.46]	5.17 [4.93, 5.45]	5.16 [4.92, 5.43]	5.13 [4.89, 5.41]	< 0.001	0.863
UA, μmol/L	265.1[228.4,306.2]	263.9[232.4,304.0]	263.8[229.8,300.0]	260.6[227.7,298.2]	0.029	0.606
BUN, mmol/L	4.83 [4.13, 5.66]	4.78 [4.08, 5.59]	4.73 [4.06, 5.50]	4.65 [4.02, 5.40]	< 0.001	< 0.001
Smoke, *n* (%)						
Non-smoking	611 (97.8)	573 (98.8)	555 (98.4)	570 (98.3)	0.579	0.644
Current	14 (2.2)	7 (1.2)	9 (1.6)	10 (1.7)		
Drunk, *n* (%)						
Non-drinking	555 (87.5)	506 (85.3)	488 (85.5)	498 (84.4)	0.449	0.193
Current	79 (12.5)	87 (14.7)	83 (14.5)	92 (15.6)		
Hypertension,* n* (%)						
No	2309 (91.7)	2164 (90.4)	2214 (92.0)	2228 (92.0)	0.124	0.389
Yes	209 (8.3)	231 (9.6)	192 (8.0)	194 (8.0)		
Diabetes, *n* (%)						
No	2472 (98.2)	2352 (98.2)	2364 (98.3)	2386 (98.5)	0.791	0.422
Yes	46 (1.8)	43 (1.8)	42 (1.7)	36 (1.5)		
Fatty liver, *n* (%)						
No	1987 (78.9)	1889 (78.9)	1963 (81.6)	2062 (85.1)	< 0.001	< 0.001
Yes	531 (21.1)	506 (21.1)	443 (18.4)	360 (14.9)		
Fundus atherosclerosis, *n* (%)						
No	2277 (90.4)	2160 (90.2)	2175 (90.4)	2206 (91.1)	0.738	0.475
Yes	241 (9.6)	235 (9.8)	231 (9.6)	216 (8.9)		

ALT, alanine aminotransferase; AST, aspartate aminotransferase; γ-GT, γ-glutamyl transpeptidase; TP, serum total protein; TG, triacylglycerol; CHOL, cholesterol; HDL, high density lipoprotein; LDL, low Density Lipoprotein; FBG, fasting blood glucose; UA, uric acid; BUN, blood urea nitrogen; IQR: interquartile range.

The incidence of fundus arteriosclerosis was 14.6%. As the TBIL level increased, the incidence of fundus arteriosclerosis gradually increased (*P* for trend < 0.001), and the number of drinkers gradually increased, but not statistically significant for smokers. Furthermore, TBIL level was positively correlated with BMI, ALT, AST, γ-GT, albumin, HDL, FBG, and UA. It was negatively correlated with BUN and globulin (Supplementary Table S1). In addition, the difference in gender composition was statistically significant among the 4 groups of TBIL level (Supplementary Table S1), and the incidence of fundus arteriosclerosis also differed in different gender groups (*P* value < 0.0001, Supplementary Figure S1).

### Comparison of the cumulative incidence of fundus arteriosclerosis in different genders

**Figure 2:**
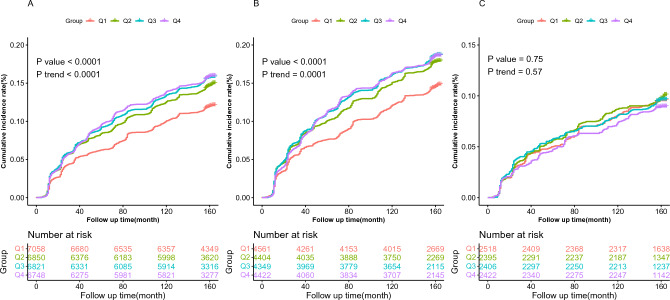
Comparison of the cumulative incidence of fundus arteriosclerosis in different genders. (**A**) Cumulative incidence curve of fundus atherosclerosis in the total population, TBIL quartile (Q1–Q4) were ≤ 10.1, 10.1–12.7, 12.7–15.9, > 15.9 μmol/L, respectively; (**B**) Cumulative incidence curve of fundus atherosclerosis in males, TBIL levels were categorized into four groups (Q1–Q4) ≤ 10.6, 10.6–13.3, 13.3–16.6, > 16.6 μmol/L for males; (**C**) Cumulative incidence curve of fundus atherosclerosis in females, TBIL levels were categorized into four groups (Q1-Q4) ≤ 9.4, 9.4–11.7, 11.7–14.4, > 14.4 μmol/L for females.

In the total population, the Q1 group (TBIL level ≤ 10.1 μmol/L) had the lowest incidence of fundus arteriosclerosis, and with the increase of TBIL level, the incidence of fundus arteriosclerosis gradually increased (*P* for trend < 0.0001, Fig. 2A). The same results were found in males (*P* for trend = 0.0001, Fig. 2B). However, in females, the incidence of fundus arteriosclerosis did not show any trend with an increase in TBIL level, and there was no statistical difference in the incidence between different groups (*P* for trend = 0.57, Fig. 2C).

### The incidence of fundus arteriosclerosis was compared among different populations

As what is shown in Table 3, after adjusting for age, BMI, FBG, smoking, drinking, AST, ALT, γ-GT, TG, TP, Albumin, Globulin, CHOL, HDL, UA, BUN, hypertension, diabetic and fatty liver status, we found that compared with group Q1 (the first quartile of TBIL level), Q2 to Q4 of TBIL in total population were risk factors for fundus arteriosclerosis. The HRs with 95% CIs were 1.240 (1.119–1.375) in Q2, 1.329 (1.200–1.471) in Q3, and 1.383 (1.248–1.534) in Q4, respectively. P value for trend was < 0.001. The same results were found in males. The HRs with 95% CIs for Q2-Q4 were 1.236 (1.112–1.374) in Q2, 1.262 (1.135–1.403) in Q3, and 1.428 (1.283–1.589) in Q4, respectively. The trend test was also significant with *P* < 0.001. Whereas, in females, we did not observe a correlation between TBIL and the development of fundus arteriosclerosis, and HRs from Q2 to Q4 were not statistically significant. The trend test was not significant with *P* = 0.057.

**Table 3:** Cox regression analysis of TBIL and fundus arteriosclerosis in different populations.

Level	Case	Model 1*		Model 2^†^		Model 3^‡^	
		HR (95% CI)	*P* value	HR (95% CI)	*P* value	HR (95% CI)	*P* value
Overall, Total Bilirubin (μmol/L, Median [IQR])							
Q1 (8.6[≤ 10.1])	847	1(Reference)		1(Reference)		1(Reference)	
Q2 (11.5[10.1–12.7])	1010	1.254(1.144–1.374)	< 0.001	1.237(1.117–1.371)	< 0.001	1.232(1.111–1.366)	< 0.001
Q3 (14.1[12.7–15.9])	1074	1.348(1.232,1.475)	< 0.001	1.337(1.210–1.478)	< 0.001	1.312(1.184–1.453)	< 0.001
Q4 (18.9[> 15.9])	1068	1.357(1.240,1.485)	< 0.001	1.387(1.256–1.532)	< 0.001	1.366(1.231–1.515)	< 0.001
*P* for trend		< 0.001		< 0.001		< 0.001	
Male, Total Bilirubin (μmol/L, Median [IQR])							
Q1 (9.0[≤ 10.6])	667	1(Reference)		1(Reference)		1(Reference)	
Q2 (12.0[10.6–13.3])	784	1.246(1.123–1.381)	< 0.001	1.241(1.117–1.378)	< 0.001	1.217(1.095–1.354)	< 0.001
Q3 (14.8[13.3–16.6])	808	1.310(1.182–1.451)	< 0.001	1.321(1.190–1.465)	< 0.001	1.255(1.128–1.396)	< 0.001
Q4 (19.6[> 16.6])	817	1.300(1.173–1.439)	< 0.001	1.431(1.289–1.587)	< 0.001	1.396(1.254–1.555)	< 0.001
*P* for trend		< 0.001		< 0.001		< 0.001	
Female, Total Bilirubin (μmol/L, Median [IQR])							
Q1 (8.0[≤ 9.4])	241	1(Reference)		1(Reference)		1(Reference)	
Q2 (10.6[9.4–11.7])	235	1.031(0.861–1.234)	0.742	1.063(0.812–1.392)	0.655	1.054(0.800–1.389)	0.707
Q3 (13.0[11.7–14.4])	231	1.010(0.843–1.210)	0.914	0.970(0.724–1.281)	0.828	1.001(0.757–1.344)	0.954
Q4 (17.075[> 14.4])	216	0.934(0.777–1.122)	0.463	1.193(0.913–1.560)	0.196	1.308(0.987–1.734)	0.062
*P* for trend		0.406		0.285		0.085	

HR and 95% CI for changes in TBIL for fundus arteriosclerosis incidence according to quartiles of TBIL in total population, male and female. *: Model1, non-adjusted; ^†^: Model2, adjusted for age, BMI, FBG, smoke, drunk; ^‡^: Model3, adjusted for age, BMI, FBG, smoke, drunk, AST, ALT, γ-GT, TG, TP, Albumin, Globulin, CHOL, HDL, LDL, UA, BUN, hypertension status, diabetes status and fatty liver status.

HR: hazard ratio; CI: confidence interval; IQR: interquartile range.

**Figure 3:**
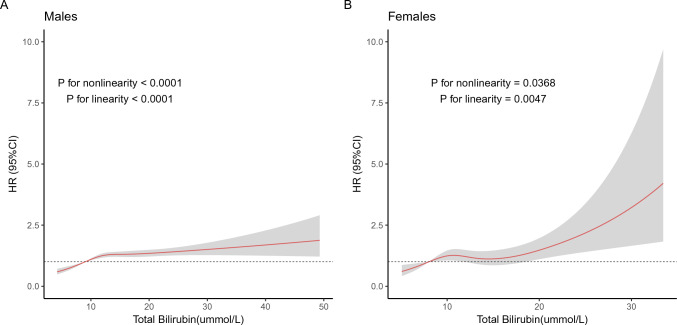
Association of TBIL with the incidence of fundus arteriosclerosis. (**A**) Restricted cubic spline plot in males; (**B**) Restricted cubic spline plot in females. According to restricted cubic spline regressions using four knots in males and females (percentiles 5, 35, 65 and 95), with the reference point set at percentile 12.5. Hazards ratios were adjusted for age, BMI, FBG, smoke, drunk, AST, ALT, γ-GT, TG, TP, Albumin, Globulin, CHOL, HDL, LDL, UA, BUN, hypertension status, diabetes status and fatty liver status, respectively. HR: hazard ratio; CI: confidence interval.

The relationship between TBIL and the incidence of fundus arteriosclerosis was established by multivariate adjusted 4-knots restricted cubic spline (RCS) regression model, suggested that there was a linear dose–response relationship between TBIL and the incidence of fundus arteriosclerosis in males (*P* < 0.0001, Fig. 3A), and with the increase of TBIL level, the risk of fundus arteriosclerosis gradually increased. In females, nonetheless, the risk of fundus arteriosclerosis was not statistically significant (*P* for Q2–Q3 was 0.656, 0.907 and 0.053; see Table 3) despite a linear dose–response relationship (*P* = 0.0047, Fig. 3B). There was also a linear dose–response relationship between TBIL and fundus arteriosclerosis in total population (*P* < 0.0001, Supplementary Figure S2).

### Association of TBIL with the incidence of fundus arteriosclerosis in the age subgroups

In two subgroups of the male (≤ 60 years and > 60 years), univariate Cox analysis showed a significant correlation between TBIL and the development of fundus atherosclerosis in three groups of Q2 to Q4, using Q1 as the reference group. This relationship was significant after adjusting to all confounding factors, and high TBIL level were risk factors for the incidence of fundus atherosclerosis (all *P* values < 0.01). Similarly, the trend test was also significant (*P* for trend < 0.001, Supplementary Table S2). In female subgroup with age ≤ 60 years, in a multivariate analysis we found that only the highest level of TBIL (Q4) was an independent risk factor for the development of fundic atherosclerosis with HR (95% CI) of 1.467 (1.058–2.033) in Q4 (*P* = 0.022), and the trend test was also significant (*P* for trend = 0.018). However, there was no significant association between TBIL and the development of fundus atherosclerosis in the subgroup with age > 60 years, and the trend test was also not significant (*P* for trend = 0.622, Supplementary Table S2). We also observed gender specificity between TBIL and fundus atherosclerosis in the age subgroup analysis.

Subsequently, in the dose–response relationship analysis of age subgroup, it was observed that there was a linear dose–response relationship between TBIL and the development of fundus arteriosclerosis in males aged ≤ 60 and > 60 years (linear *P* values < 0.0001, Supplementary Figure S3A,B), and a progressive increase in the risk of developing fundus arteriosclerosis with increasing TBIL level. Moreover, there was a linear dose–response relationship in females with age ≤ 60 years (*P* = 0.0039). Conversely, there was no linear dose–response relationship in females with age > 60 years (*P* = 0.6042) (Supplementary Figure S3C,D).

### Association of TBIL with the incidence of fundus arteriosclerosis in the BMI subgroups

In the same way, we observed the same gender-specific association for the subgroup analysis of BMI. In male with a BMI of 25 kg/m^2^ or less, after adjusting for potential confounders, the Q3 and Q4 of TBIL level were risk factors for fundus arteriosclerosis. The HRs (95% CIs) was 1.233 (1.053–1.444) in Q3 and 1.320 (1.124–1.551) in Q4. The trend test was also significant (*P* for trend < 0.001). In male subgroup with > 25 kg/m^2^, the Q2, Q3 and Q4 of TBIL were risk factors for fundus arteriosclerosis (all *P* values < 0.001). Similarly, the trend test was also significant (*P* for trend < 0.001, Supplementary Table S3). This suggested that the risk of developing fundus atherosclerosis increases with increasing TBIL. In both females with BMI ≤ 25 kg/m^2^ and BMI > 25 kg/m^2^ subgroups, we could find that there were no statistically significant differences between the Q2 to Q4 of TBIL (Q1 as reference) and the risk of incident fundus atherosclerosis, and the trend test was not significant with 0.133 and 0.906, respectively (Supplementary Table S3).

The dose–response relationships analysis revealed that there was a linear dose–response relationship between TBIL and incident fundus atherosclerosis in males with BMI ≤ 25 kg/m^2^ and in those with BMI > 25 kg/m^2^ (*P* = 0.0003 and *P* < 0.0001). In females, there also was a linear dose–response relationship between TBIL and fundus atherosclerosis in the subgroup with BMI ≤ 25 kg/m^2^ (*P* = 0.0242), whereas there was no linear dose–response relationship between the subgroup with BMI > 25 kg/m^2^ (*P* = 0.4557, Supplementary Figure S4).

### Association of TBIL with the incidence of fundus arteriosclerosis in the hypertension subgroups

After adjusting to potential confounders, the relationship between TBIL and incident fundus arteriosclerosis by hypertension subgroup was found that the highest level of TBIL (Q4) was an independent risk factor for fundus arteriosclerosis, in males with hypertension. The HR (95% CI) was 1.253 (1.063–1.475) with *P* = 0.007. In males without hypertension, from the second to the fourth quartiles of TBIL levels, the HRs (95% CIs) in Q2 was 1.371 (1.195–1.574), in Q3 was 1.424 (1.241–1.633), and in Q4 was 1.545 (1.342–1.779), the trend test was also significant (*P* for trend < 0.001) (Supplementary Table S4). This means that high level of TBIL was a risk factor for the development of fundus atherosclerosis, and the risk increases with higher TBIL. Furthermore, in the dose–response relationship analysis of hypertension subgroups, we could observe that there was a linear dose–response relationship between TBIL and the incidence of fundus arteriosclerosis in males with hypertension and without hypertension (linear *P* values < 0.05, Supplementary Figure S5A,B). Notably, in males without hypertension, the risk of developing fundus atherosclerosis gradually decreased when TBIL levels exceeded 19.27 μmol/L.

In females with and without hypertension, we were unable to find any statistically significant differences between the Q2 to Q4 of TBIL and the risk of developing fundus atherosclerosis, and the trend test was not significant, with *P* values of 0.219 and 0.469, respectively (Supplementary Table S4). The results of the dose–response relationship suggested that there were no linear dose–response relationships between TBIL and fundus atherosclerosis in both subgroups of females, with linear *P* values of 0.1790 and 0.1154, respectively (Supplementary Figure S5C,D).

### Association of TBIL with the incidence of fundus arteriosclerosis in the diabetes subgroups

In like manner, the relationship between TBIL and incident fundus arteriosclerosis by diabetes subgroup analysis in subgroup analysis. We observed that in males with diabetes subgroup, after adjusting for potential confounders, the Q4 of TBIL was an independent risk factor for fundus arteriosclerosis, The HR (95% CI) was 1.356 (1.011–1.820) with *P* = 0.042. In males without diabetes, from the second to the fourth quartile of TBIL, the HRs (95% CIs) was 1.284 (1.147–1.439) in Q2, 1.372 (1.226–1.535) in Q3, 1.404 (1.251–1.576) in Q4, respectively. The trend test was also significant with *P* < 0.001 (Supplementary Table S5. This indicated that the risk of developing fundus atherosclerosis increases with increasing TBIL. In both females with and without diabetes, we were able to find no statistically significant differences between the Q2–Q4 of TBIL, and the risk of incident fundus atherosclerosis, *P* for trend = 0.631 and 0.350, respectively.

Subsequently, the dose–response relationships analysis in the diabetes subgroup showed that there was a linear dose–response relationship between TBIL and fundus atherosclerosis in males with and without diabetes (*P* = 0.0042 and *P* < 0.0001, Supplementary Figure S6A,B). In females, there was a linear dose–response relationship between TBIL of fundus atherosclerosis in the subgroup without diabetes (*P* = 0.0214), whereas there was no linear dose–response relationship of the subgroup with diabetes (*P* = 0.1992, Supplementary Figure S6C,D).

## Discussion

The results of this study showed that in the general population, the incidence of fundus atherosclerosis was 14.6%, including 17.3% in men and 9.5% in women. Compared with other studies^30,31^, the incidence of fundus arteriosclerosis in our study was slightly higher, which should be highly concerned. The main possible reasons are the differences in research design and research objects. Secondly, hypertensive, diabetic, and fatty liver patients were not excluded from the population screening in this study, which could also account for the higher incidence of fundus arteriosclerosis. In this study, we found a positive correlation between the incidence of fundus arteriosclerosis and serum TBIL level. Our study showed that, after adjusting for other potential confounding risk factors, elevated TBIL was an independent risk factor for fundus arteriosclerosis in men. However, these findings were not significant in females. In the subgroup analysis, we likewise observed a gender-specific association between them. In addition, there was a linear dose–response relationship between total bilirubin level and the incidence of fundus arteriosclerosis.

Our findings conflict with the conclusions of most existing studies. At present, the mainstream studies are focused on diabetic retinopathy, but there is still a lack of research on fundus arteriosclerosis. In a 5-year cohort study involving 5323 Chinese men with diabetes^32^, they found a U-shaped relationship between serum TBIL and the development of diabetic retinopathy, representing a protective relationship at lower level and a risk factor at higher level. Our study also found that at high level, TBIL was a risk factor for fundus arteriosclerosis. In a cross-sectional study conducted by Liu et al.^33^, it was found that TBIL were inversely associated with diabetic complications in an elderly diabetic population, and that the incidence of retinopathy was significantly reduced with increased TBIL, and that high TBIL level was a protective factors for the development of retinopathy. In addition, a strong inverse association between serum TBIL level and diabetic retinopathy and nephropathy was identified in a Japanese population without severe liver dysfunction, and low TBIL level are a risk factor for diabetic retinopathy in Japanese patients with type 2 diabetes^34^. In a longitudinal study by Chen et al.^35^, TBIL was grouped by quintile, and the results showed that higher TBIL level effectively reduced the risk of cardiovascular disease and all-cause death in diabetic patients, improved the risk prediction of cardiovascular death, and had better predictive ability. The findings of these studies differ from the conclusions of our study, probably because they studied mostly people with diabetes, while our study was the general population, not just with diabetes. It is well known that diabetic retinopathy, like fundus arteriosclerosis, is a microvascular disease of the eye. The use of some anti-diabetic drugs, such as angiotensin 2 receptor antagonists^34^, can affect oxidative stress and play an antioxidant role, so it may have a protective effect on vascular atherosclerosis.

Fundus arteriosclerosis is an atherosclerotic disease of small arteries in the eye, which can reflect small vessel disease in the brain and large artery atherosclerotic disease in the whole body. In our study, the incidence of fundus arteriosclerosis was higher in men in the fourth quartile than in the other three groups. Moreover, after adjusting for potential confounding factors, high level of TBIL is a risk factor for fundus arteriosclerosis. However, the biological mechanism behind this association, which was found only in the male group but not in the female group, is not clear. It is well known that atherosclerosis is an inflammatory disease in which lipid deposition in the arterial wall due to elevated plasma cholesterol level is central to the development of lesions^36^. This process involves the uptake of modified LDL cholesterol by macrophages and is associated with changes in oxidative stress and damage status^37^. HDL, a known protective factor for atherosclerosis, showed a positive correlation with high TBIL at baseline in the study. This correlation may be due to some basic characteristics as confounding factors. After adjustment, HDL was an independent protective factor for fundus arteriosclerosis in total population and men. But there was no association among women (Supplementary Tables S7, S8and S9).

Most studies have shown that bilirubin plays an important antioxidant role in the protection of atherosclerosis. Bilirubin is an effective scavenger of oxidants in vitro. For example, when micromolar concentrations of bilirubin are added to cell culture medium, it protects various cells, including endothelial cells and smooth muscle cells, from hydrogen peroxi-induced toxicity^38^. Moreover, in addition to affecting the inflammatory process, bilirubin can also prevent neointimal formation by inhibiting smooth muscle cell proliferation. It has been shown that local administration of bilirubin reduces neointimal formation and regulates the proliferation and migration of arterial smooth muscle cells after carotid artery injury in rats^39^. At the same time, bilirubin also has the effect of inhibiting endothelial dysfunction^20,40^. The results of a pilot study suggest that bilirubin can prevent endothelial dysfunction caused by oxidative stress^40^. In this study, treatment of endothelial cells with hypochlorous acid stimulated mitochondrial dysfunction and cell death, effects that were reversed when bilirubin was administered. These results suggest that bilirubin can maintain endothelial activity at sites of vascular inflammation and atherosclerosis. Combined with the above biological mechanisms, it is not difficult to conclude that there is a correlation between serum bilirubin and arteriosclerosis, and high level of bilirubin present a protective factor. However, it is important to note that this mechanism is only true in patients with Gilbert’s syndrome. In addition, serum bilirubin has been suggested to playing a dual role in cells, that is, at high concentrations, bilirubin is a highly cytotoxic metabolite that can cause brain damage, while at low concentrations, it is an endogenous antioxidant^20^. Other studies have shown a U-shaped relationship between TBIL and cardiovascular disease^41^. In a review study it was proposed that high bilirubin level due to underlying abnormal liver function were not protective against cardiovascular disease^42^. Chen’s study also showed that a higher TBIL level was positively associated with an increased risk of all-cause mortality, and the fourth quartile bilirubin level was a risk factor for all-cause mortality risk^43^.

We also examined the association between serum bilirubin and the incidence of fundus arteriosclerosis in a population with Gilbert's syndrome (benign hyperbilirubinemia, characterized by a mildly elevated serum unconjugated bilirubin level)^42^. We selected, by definition^44^, unconjugated bilirubin (indirect bilirubin) levels ≥ 17.1 μmol/L of 931 (3.4%) were analyzed as Gilbert's syndrome population. There were 743 males (79.8%) and 188 females (20.2%). Similarly, the first quartile of TBIL was used as the reference to evaluate the hazard ratio (HR) between TBIL quartile (Q2–Q4) and fundus arteriosclerosis. Our results found that the incidence of fundus arteriosclerosis was 20.1% in the total population, 22.3% in men and 11.2% in women. The results of univariate Cox analysis showed that in the total population, male and female population, the high level of TBIL was a protective factor for fundus arteriosclerosis (*P* value < 0.05, *P* for trend < 0.05) (Supplementary Table S6). After adjusting to confounders, we found that compared with Q1 group, Q2 and Q4 of TBIL level are protective factors for fundus arteriosclerosis in males. The HRs with 95% CIs were 0.598 (0.382–0.935) in Q2, 0.760 (0.493–1.171) in Q3, and 0.682 (0.418–0.911) in Q4, respectively (*P* for trend = 0.263). The sample size for women was insufficient to calculate the HR and *P* value. Our findings indicate that there is an inverse correlation between high TBIL level and fundus arteriosclerosis in male, and that high TBIL is a protective factor for fundus arteriosclerosis in patients with underlying Gilbert's syndrome. The results of our study are consistent with the conclusions of the study by L. VITEK^42^. It follows that bilirubin, as an endogenous antioxidant, plays a key role in populations with benign hyperbilirubinemia and should be of concern. In addition, we analyzed the association between DBIL and IBIL and fundus atherosclerosis in three populations separately (Supplementary Tables S10 and S11). We found that DBIL was a protective factor for fundus atherosclerosis (consistent with the findings of Wan et al.^15^) and IBIL was a risk factor for fundus atherosclerosis, and neither of them was gender-specific.

As a consequence, the statistical and biological correlation between serum total bilirubin and fundus arteriosclerosis cannot be generalized. It can be roughly summarized that when bilirubin is high in benign blood in men, high level of TBIL protect against cardiovascular disease. secondly when men because of the potential of abnormal liver function and cause high serum bilirubin, there were no protective effect on cardiovascular function. When the level of hyperbilirubinemia is normal in men and other related liver function diseases are not considered, hyperbilirubinemia is a risk factor for retinal artery. Whether it is a risk factor for other cardiovascular diseases needs to be further studied in related population experiments.

Our study also has some limitations. First, we had difficulties in screening the cohort because we could not detect the presence of abnormal liver function in the individuals recorded in the database due to the early time of data collection. Second, we did not exclude people with diabetes, hypertension, and abnormal liver function, which are potential contributors to fundus arteriosclerosis. Nevertheless, the strengths of our study are the large sample size and long cohort study. We analyzed men and women separately, which ensured that the parameters were adequate and the results were accurate, and we came to a reliable conclusion that there were differences between men and women. Finally, we will examine the relationship between serum TBIL and fundus arteriosclerosis in a healthy population and determine whether bilirubin levels (both direct and indirect bilirubin) can improve the prediction of fundus arteriosclerosis.

## Conclusion

This study sought to investigate the association between total serum bilirubin and the incidence of fundus arteriosclerosis using multivariate Cox models in a Chinese medical examination cohort population.

- The study revealed a 14.6% prevalence of fundus atherosclerosis, which was positively associated with TBIL levels.
- After adjusting for potential confounders, the TBIL was an independent risk factor for fundus atherosclerosis in men. The association between TBIL and fundus atherosclerosis became stronger with higher TBIL levels, and the risk of developing fundus atherosclerosis increased. Conversely, these findings were not statistically significant in the female population.
- In subgroup analyses of age, BMI, hypertension, and diabetes populations, we similarly observed gender differences between TBIL and fundus atherosclerosis.
- There was a linear dose–response relationship between TBIL levels and the incidence of fundus atherosclerosis.

## Supplementary Information

Supplementary Information.

## Data Availability

The datasets generated during and/or analyzed during the current study are not publicly available due to cooperation unit needs to be confidential, but are available from the corresponding author on reasonable request.
