# Supplementary material for: Association between total bilirubin and gender-specific incidence of fundus arteriosclerosis in a Chinese population: a retrospective cohort study
Source: Sci Rep. 2023 Jul 11;13:11244. doi: 10.1038/s41598-023-38378-1 (PMC10336033; doi:10.1038/s41598-023-38378-1)
Supplement: Supplementary file 1 — Supplementary Information. [file 41598_2023_38378_MOESM1_ESM.doc]

**Supplementary files**

**Legends：**

**Supplementary Figure S1 Cumulative incidence curves between gender and occurrence of fundus arteriosclerosis in the total population.**

**Supplementary Figure S2 Association of TBIL with the incidence of fundus arteriosclerosis in total population.** According to restricted cubic spline regressions using four knots in total population (percentiles 5, 35, 65 and 95), with the reference point set at percentile 12.5. Hazards ratios were adjusted for age, BMI, FBG, smoke, drunk, AST, ALT, γ-GT, TG, TP, Albumin, Globulin, CHOL, HDL, LDL, UA, BUN, hypertension status, diabetes status and fatty liver status, respectively.

HR: hazard ratio; CI: confidence interval.

**Supplementary Figure S3** **Association of TBIL with the incidence of fundus arteriosclerosis in the age subgroups.** A: Restricted cubic spline plot in male subgroup with age ≤ 60 years; B: Restricted cubic spline plot in male subgroup with age > 60 years. C: Restricted cubic spline plot in female subgroup with age ≤ 60 years; D: Restricted cubic spline plot in female subgroup with age > 60 years. According to restricted cubic spline regressions using four knots in males and females (percentiles 5, 35, 65 and 95), with the reference point set at percentile 12.5. Hazards ratios were adjusted for age, BMI, FBG, smoke, drunk, AST, ALT, γ-GT, TG, TP, Albumin, Globulin, CHOL, HDL, LDL, UA, BUN, hypertension status, diabetes status and fatty liver status, respectively.

HR: hazard ratio; CI: confidence interval.

**Supplementary Figure S4** **Association of TBIL with the incidence of fundus arteriosclerosis in the BMI subgroups.** A: Restricted cubic spline plot in male subgroup with BMI ≤ 25 kg/m^2^; B: Restricted cubic spline plot in male subgroup with BMI > 25 kg/m^2^. C: Restricted cubic spline plot in female subgroup with BMI ≤ 25 kg/m^2^; D: Restricted cubic spline plot in female subgroup with BMI > 25 kg/m^2^. According to restricted cubic spline regressions using four knots in males and females (percentiles 5, 35, 65 and 95), with the reference point set at percentile 12.5. Hazards ratios were adjusted for age, BMI, FBG, smoke, drunk, AST, ALT, γ-GT, TG, TP, Albumin, Globulin, CHOL, HDL, LDL, UA, BUN, hypertension status, diabetes status and fatty liver status, respectively.

HR: hazard ratio; CI: confidence interval.

**Supplementary Figure S5** **Association of TBIL with the incidence of fundus arteriosclerosis in the hypertension subgroups.** A: Restricted cubic spline plot in male subgroup with hypertension; B: Restricted cubic spline plot in male subgroup without hypertension. C: Restricted cubic spline plot in female subgroup with hypertension; D: Restricted cubic spline plot in female subgroup without hypertension. According to restricted cubic spline regressions using four knots in males and females (percentiles 5, 35, 65 and 95), with the reference point set at percentile 12.5. Hazards ratios were adjusted for age, BMI, FBG, smoke, drunk, AST, ALT, γ-GT, TG, TP, Albumin, Globulin, CHOL, HDL, LDL, UA, BUN, hypertension status, diabetes status and fatty liver status, respectively.

HR: hazard ratio; CI: confidence interval.

**Supplementary Figure S6** **Association of TBIL with the incidence of fundus arteriosclerosis in the diabetes subgroups.** A: Restricted cubic spline plot in male subgroup with diabetes; B: Restricted cubic spline plot in male subgroup without diabetes. C: Restricted cubic spline plot in female subgroup with diabetes; D: Restricted cubic spline plot in female subgroup without diabetes. According to restricted cubic spline regressions using four knots in males and females (percentiles 5, 35, 65 and 95), with the reference point set at percentile 12.5. Hazards ratios were adjusted for age, BMI, FBG, smoke, drunk, AST, ALT, γ-GT, TG, TP, Albumin, Globulin, CHOL, HDL, LDL, UA, BUN, hypertension status, diabetes status and fatty liver status, respectively.

HR: hazard ratio; CI: confidence interval.

**Supplementary Table S1 Baseline characteristics of participants in total populations**

Abbreviations: ALT, alanine aminotransferase; AST, aspartate aminotransferase; γ-GT, γ-glutamyl transpeptidase; TP, serum total protein; TG, triacylglycerol; CHOL, cholesterol; HDL, high density lipoprotein; LDL, low Density Lipoprotein; FBG, fasting blood glucose; UA, uric acid; BUN, blood urea nitrogen; IQR: interquartile range.

**Supplementary Table S2 Cox regression analysis of TBIL and fundus arteriosclerosis in different populations in age subgroups**

**Supplementary Table S3 Cox regression analysis of TBIL and fundus arteriosclerosis in different populations in BMI subgroups**

In the age subgroups, univariate and multivariate Cox regression analysis of the relationship between total bilirubin and fundus arteriosclerosis, in males and females. ^*^: Model1, non-adjusted; ^†^: Model2, adjusted for age, BMI, FBG, smoke, drunk; ^‡^: Model3, adjusted for age, BMI, FBG, smoke, drunk, AST, ALT, γ-GT, TG, TP, Albumin, Globulin, CHOL, HDL, LDL, UA, BUN, hypertension status, diabetes status and fatty liver status.

HR: hazard ratio; CI: confidence interval; IQR: interquartile range.

**Supplementary Table S4 Cox regression analysis of TBIL and fundus arteriosclerosis in different populations in hypertension subgroups**

In the BMI subgroups, univariate and multivariate Cox regression analysis of the relationship between total bilirubin and fundus arteriosclerosis, in males and females. ^*^: Model1, non-adjusted; ^†^: Model2, adjusted for age, BMI, FBG, smoke, drunk; ^‡^: Model3, adjusted for age, BMI, FBG, smoke, drunk, AST, ALT, γ-GT, TG, TP, Albumin, Globulin, CHOL, HDL, LDL, UA, BUN, hypertension status, diabetes status and fatty liver status.

HR: hazard ratio; CI: confidence interval; IQR: interquartile range.

**Supplementary Table S5 Cox regression analysis of TBIL and fundus arteriosclerosis in different populations in diabetes subgroups**

In the diabetes subgroups, univariate and multivariate Cox regression analysis of the relationship between total bilirubin and fundus arteriosclerosis, in males and females. ^*^: Model1, non-adjusted; ^†^: Model2, adjusted for age, BMI, FBG, smoke, drunk; ^‡^: Model3, adjusted for age, BMI, FBG, smoke, drunk, AST, ALT, γ-GT, TG, TP, Albumin, Globulin, CHOL, HDL, LDL, UA, BUN, hypertension status, diabetes status and fatty liver status.

HR: hazard ratio; CI: confidence interval; IQR: interquartile range.

**Supplementary Table S6 Cox regression analysis of TBIL and fundus arteriosclerosis in Gilbert’s population**

HR and 95% CI for changes in TBIL for fundus arteriosclerosis incidence according to quartiles of TBIL in total population, male and female. Notes: in women, the sample size was too small to be calculated. ^*^: Model1, non-adjusted; ^†^: Model2, adjusted for age, BMI, FBG, smoke, drunk; ^‡^: Model3, adjusted for age, BMI, FBG, smoke, drunk, AST, ALT, γ-GT, TG, TP, Albumin, Globulin, CHOL, HDL, LDL, UA, BUN, hypertension status, diabetes status and fatty liver status.

HR: hazard ratio; CI: confidence interval; IQR: interquartile range.

**Supplementary Table S7 Cox regression analysis of TBIL and fundus arteriosclerosis in total population**

HR and 95% CI for changes in TBIL for fundus arteriosclerosis incidence according to quartiles of TBIL in total population, male and female. ^*^: Model1, non-adjusted; ^†^: Model2, adjusted for gender, age, BMI, FBG, smoke, drunk; ^‡^: Model3, adjusted for gender, age, BMI, FBG, smoke, drunk, AST, ALT, γ-GT, TG, TP, Albumin, Globulin, CHOL, HDL, LDL, UA, BUN, hypertension status, diabetes status and fatty liver status.

HR: hazard ratio; CI: confidence interval; IQR: interquartile range.

**Supplementary Table S8 Cox regression analysis of TBIL and fundus arteriosclerosis in males**

HR and 95% CI for changes in TBIL for fundus arteriosclerosis incidence according to quartiles of TBIL in total population, male and female. ^*^: Model1, non-adjusted; ^†^: Model2, adjusted for age, BMI, FBG, smoke, drunk; ^‡^: Model3, adjusted for age, BMI, FBG, smoke, drunk, AST, ALT, γ-GT, TG, TP, Albumin, Globulin, CHOL, HDL, LDL, UA, BUN, hypertension status, diabetes status and fatty liver status.

HR: hazard ratio; CI: confidence interval; IQR: interquartile range.

**Supplementary Table S9 Cox regression analysis of TBIL and fundus arteriosclerosis in females**

HR and 95% CI for changes in TBIL for fundus arteriosclerosis incidence according to quartiles of TBIL in total population, male and female. ^*^: Model1, non-adjusted; ^†^: Model2, adjusted for age, BMI, FBG, smoke, drunk; ^‡^: Model3, adjusted for age, BMI, FBG, smoke, drunk, AST, ALT, γ-GT, TG, TP, Albumin, Globulin, CHOL, HDL, LDL, UA, BUN, hypertension status, diabetes status and fatty liver status.

HR: hazard ratio; CI: confidence interval; IQR: interquartile range.

**Supplementary Table S10 Cox regression analysis of DBIL and fundus arteriosclerosis in different populations**

HR and 95% CI for changes in DBIL for fundus arteriosclerosis incidence according to quartiles of TBIL in total population, male and female. ^*^: Model1, non-adjusted; ^†^: Model2, adjusted for age, BMI, FBG, smoke, drunk; ^‡^: Model3, adjusted for age, BMI, FBG, smoke, drunk, AST, ALT, γ-GT, TG, TP, Albumin, Globulin, CHOL, HDL, LDL, UA, BUN, hypertension status, diabetes status and fatty liver status.

HR: hazard ratio; CI: confidence interval; IQR: interquartile range.

**Supplementary Table S11 Cox regression analysis of IBIL and fundus arteriosclerosis in different populations**

HR and 95% CI for changes in IBIL for fundus arteriosclerosis incidence according to quartiles of TBIL in total population, male and female. ^*^: Model1, non-adjusted; ^†^: Model2, adjusted for age, BMI, FBG, smoke, drunk; ^‡^: Model3, adjusted for age, BMI, FBG, smoke, drunk, AST, ALT, γ-GT, TG, TP, Albumin, Globulin, CHOL, HDL, LDL, UA, BUN, hypertension status, diabetes status and fatty liver status.

HR: hazard ratio; CI: confidence interval; IQR: interquartile range.


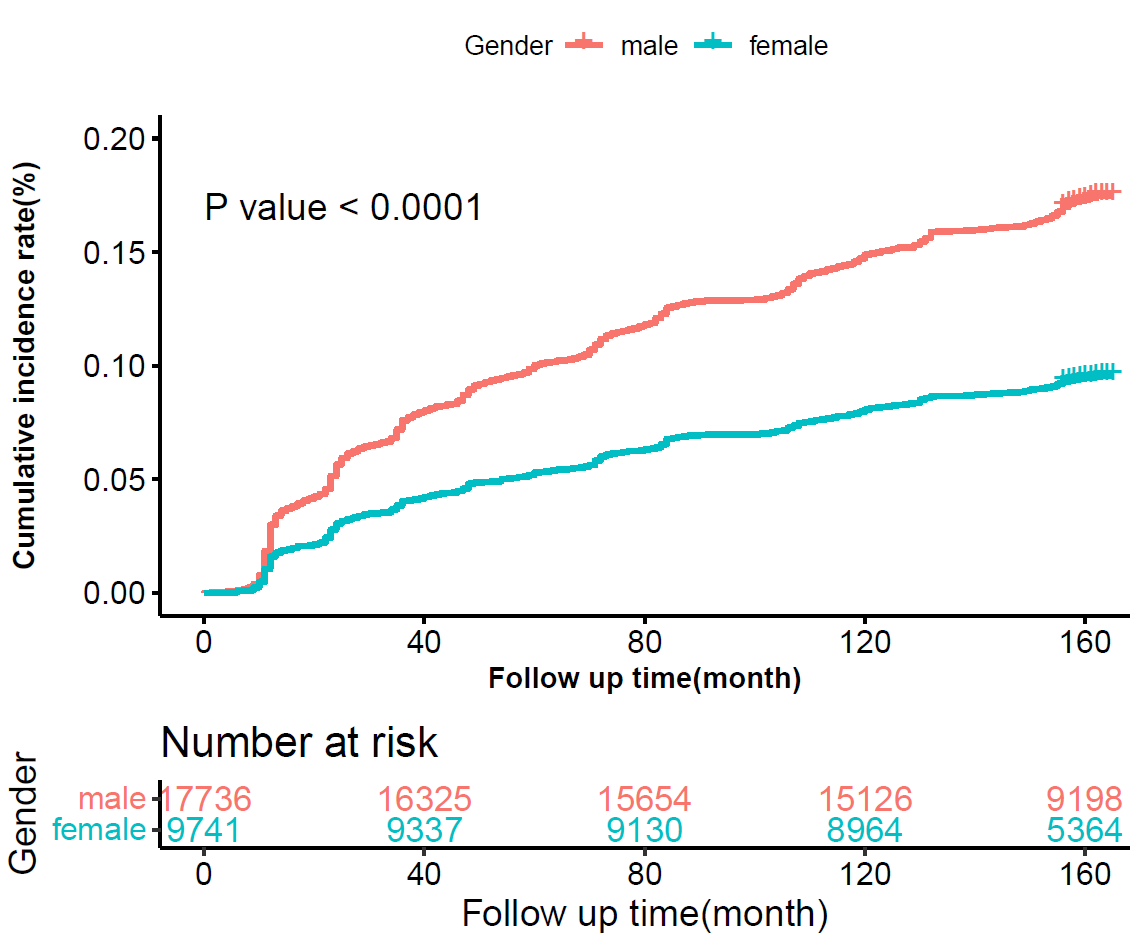


**Supplementary Figure S1 Cumulative incidence curves between gender and occurrence of fundus arteriosclerosis in the total population.**


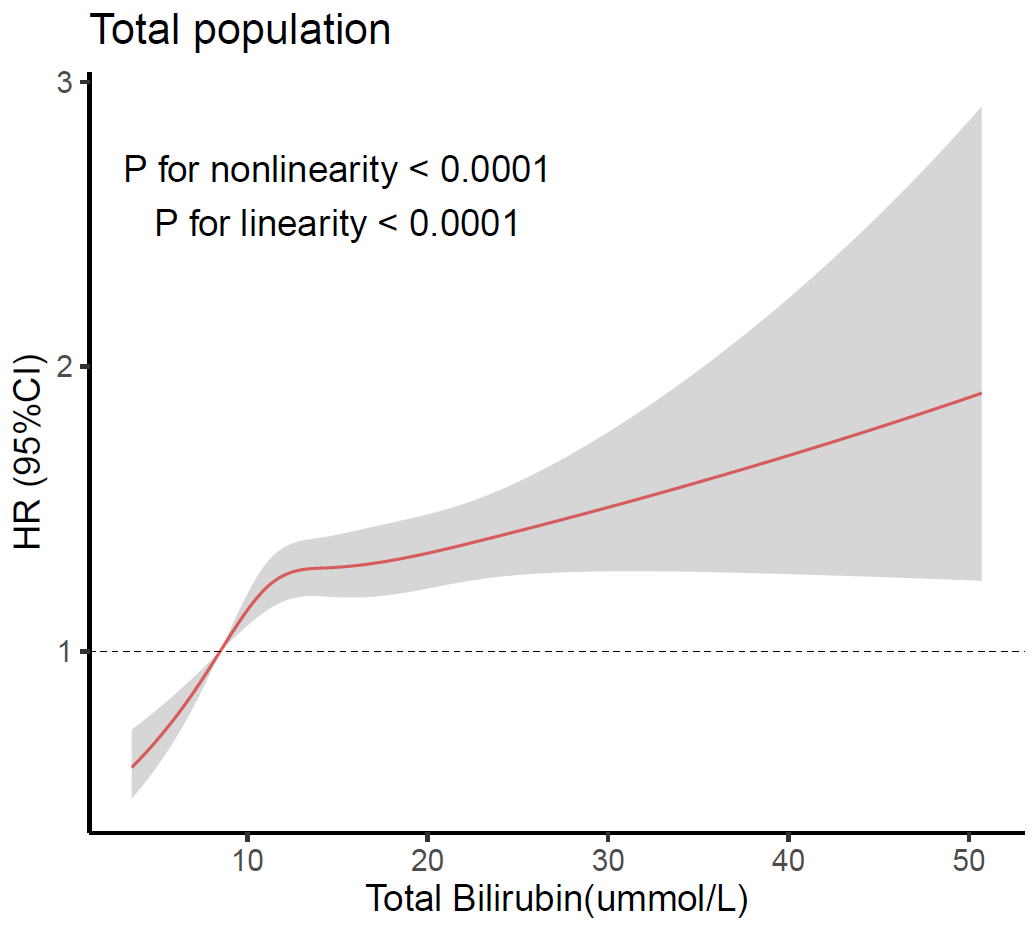


**Supplementary Figure S2 Association of TBIL with the incidence of fundus arteriosclerosis in total population.** According to restricted cubic spline regressions using four knots in total population (percentiles 5, 35, 65 and 95), with the reference point set at percentile 12.5. Hazards ratios were adjusted for age, BMI, FBG, smoke, drunk, AST, ALT, γ-GT, TG, TP, Albumin, Globulin, CHOL, HDL, LDL, UA, BUN, hypertension status, diabetes status and fatty liver status, respectively.

HR: hazard ratio; CI: confidence interval.

**
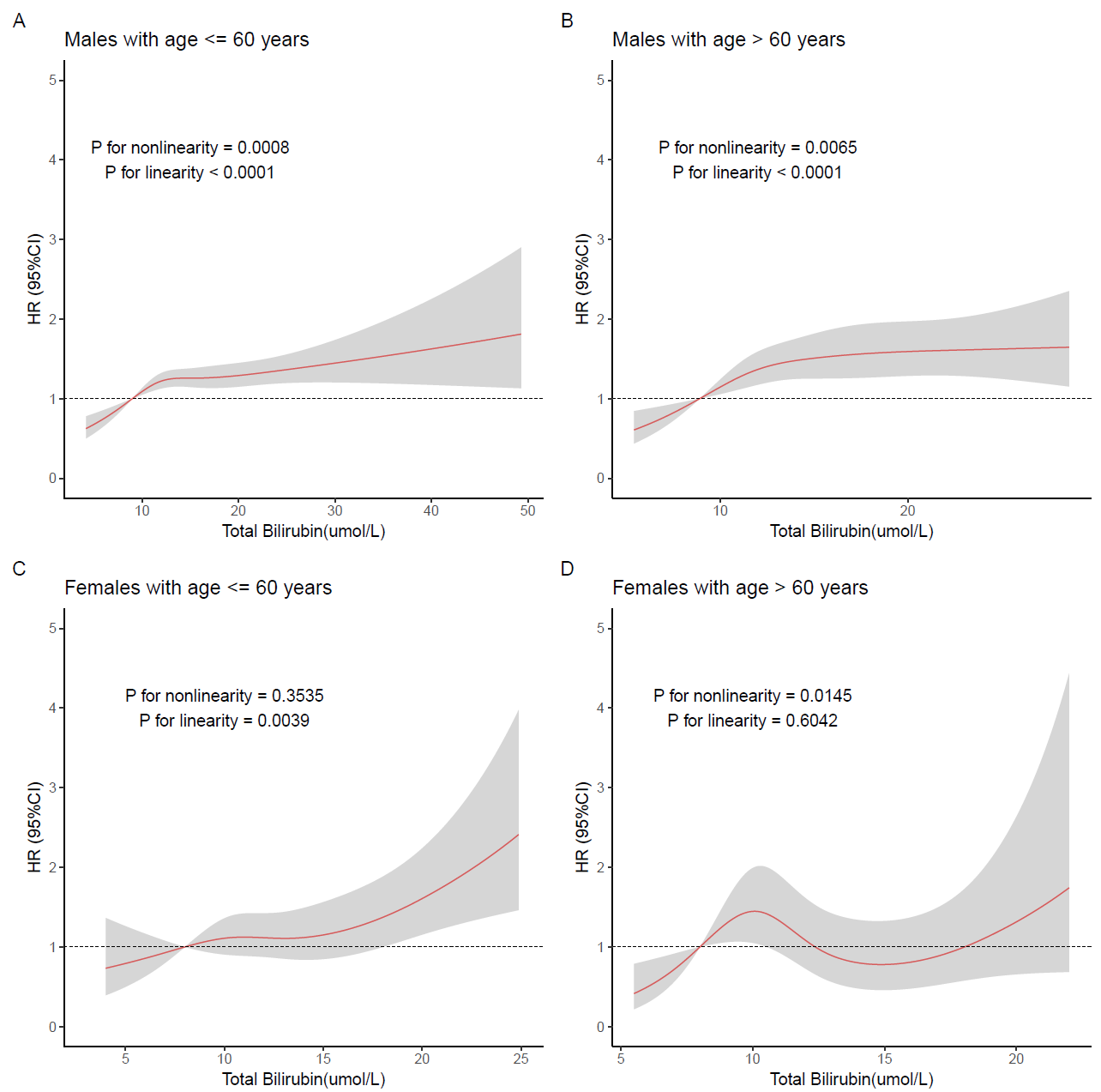
**

**Supplementary Figure S3** **Association of TBIL with the incidence of fundus arteriosclerosis in the age subgroups.** A: Restricted cubic spline plot in male subgroup with age ≤60 years; B: Restricted cubic spline plot in male subgroup with age >60 years. C: Restricted cubic spline plot in female subgroup with age ≤60 years; D: Restricted cubic spline plot in female subgroup with age >60 years. According to restricted cubic spline regressions using four knots in males and females (percentiles 5, 35, 65 and 95), with the reference point set at percentile 12.5. Hazards ratios were adjusted for age, BMI, FBG, smoke, drunk, AST, ALT, γ-GT, TG, TP, Albumin, Globulin, CHOL, HDL, LDL, UA, BUN, hypertension status, diabetes status and fatty liver status, respectively.

HR: hazard ratio; CI: confidence interval.

**
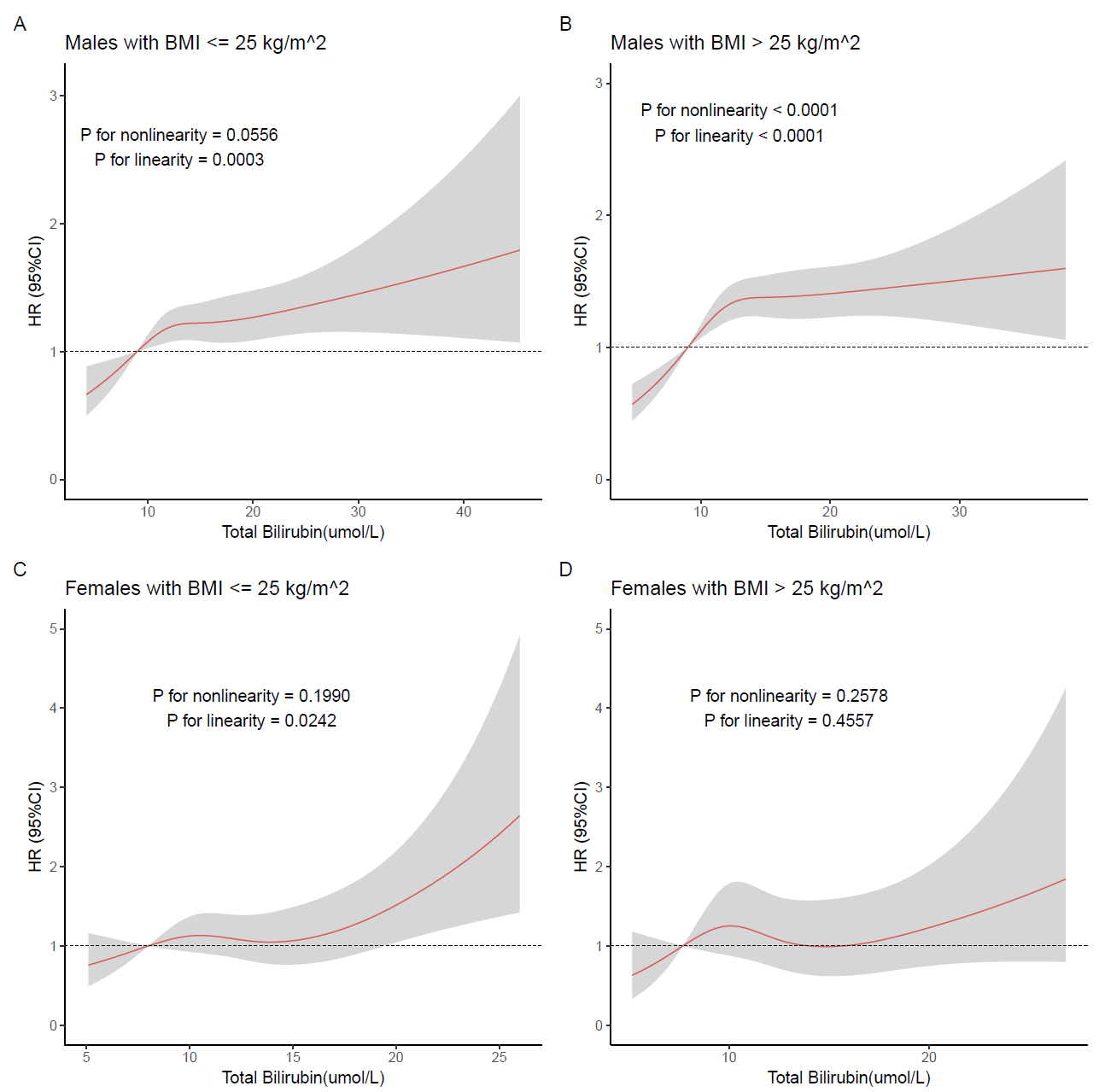
**

**Supplementary Figure S4** **Association of TBIL with the incidence of fundus arteriosclerosis in the BMI subgroups.** A: Restricted cubic spline plot in male subgroup with BMI ≤25 kg/m^2^; B: Restricted cubic spline plot in male subgroup with BMI >25 kg/m^2^. C: Restricted cubic spline plot in female subgroup with BMI ≤25 kg/m^2^; D: Restricted cubic spline plot in female subgroup with BMI >25 kg/m^2^. According to restricted cubic spline regressions using four knots in males and females (percentiles 5, 35, 65 and 95), with the reference point set at percentile 12.5. Hazards ratios were adjusted for age, BMI, FBG, smoke, drunk, AST, ALT, γ-GT, TG, TP, Albumin, Globulin, CHOL, HDL, LDL, UA, BUN, hypertension status, diabetes status and fatty liver status, respectively.

HR: hazard ratio; CI: confidence interval.

**
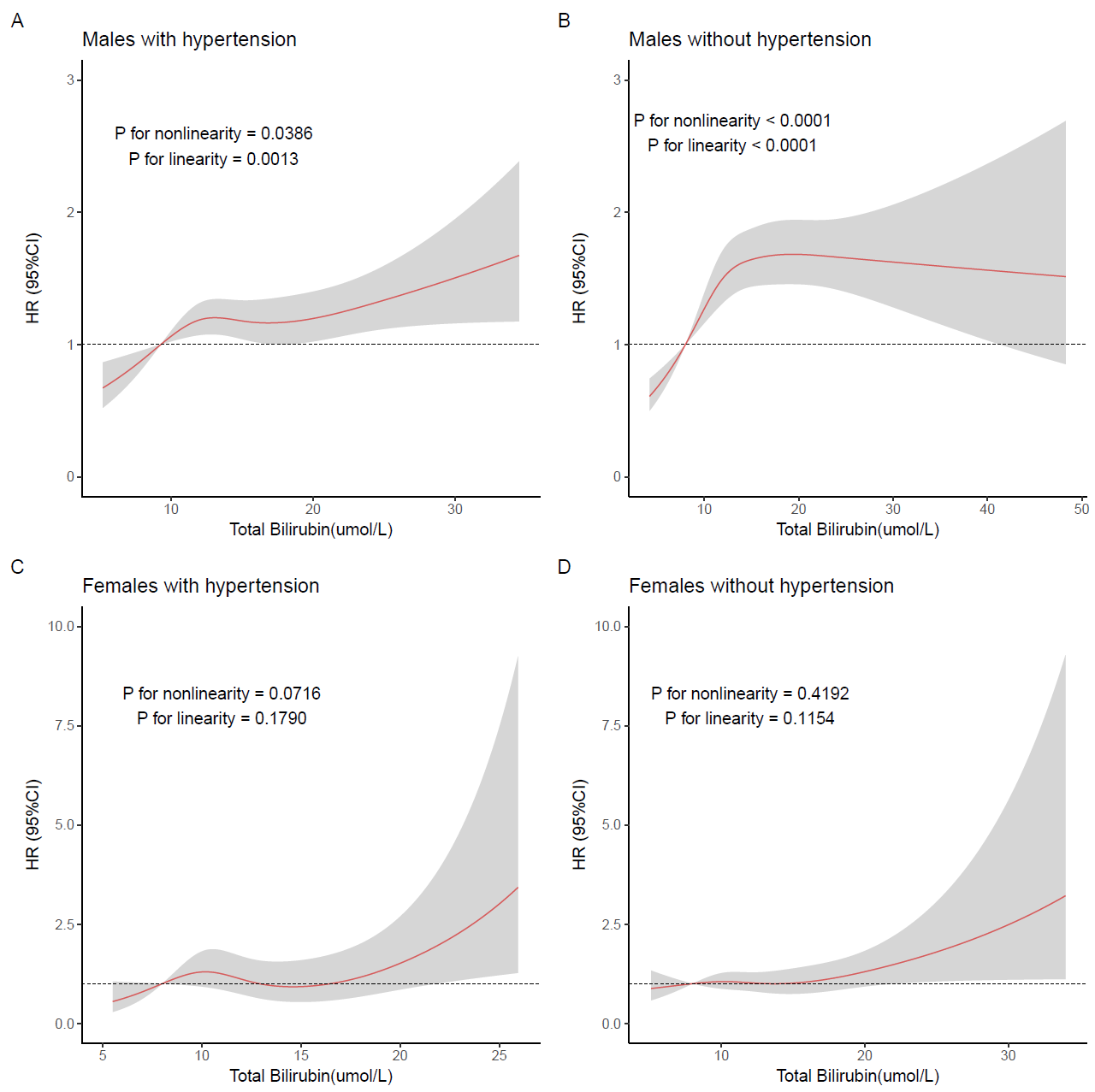
**

**Supplementary Figure S5** **Association of TBIL with the incidence of fundus arteriosclerosis in the hypertension subgroups.** A: Restricted cubic spline plot in male subgroup with hypertension; B: Restricted cubic spline plot in male subgroup without hypertension. C: Restricted cubic spline plot in female subgroup with hypertension; D: Restricted cubic spline plot in female subgroup without hypertension. According to restricted cubic spline regressions using four knots in males and females (percentiles 5, 35, 65 and 95), with the reference point set at percentile 12.5. Hazards ratios were adjusted for age, BMI, FBG, smoke, drunk, AST, ALT, γ-GT, TG, TP, Albumin, Globulin, CHOL, HDL, LDL, UA, BUN, hypertension status, diabetes status and fatty liver status, respectively.

HR: hazard ratio; CI: confidence interval.

**
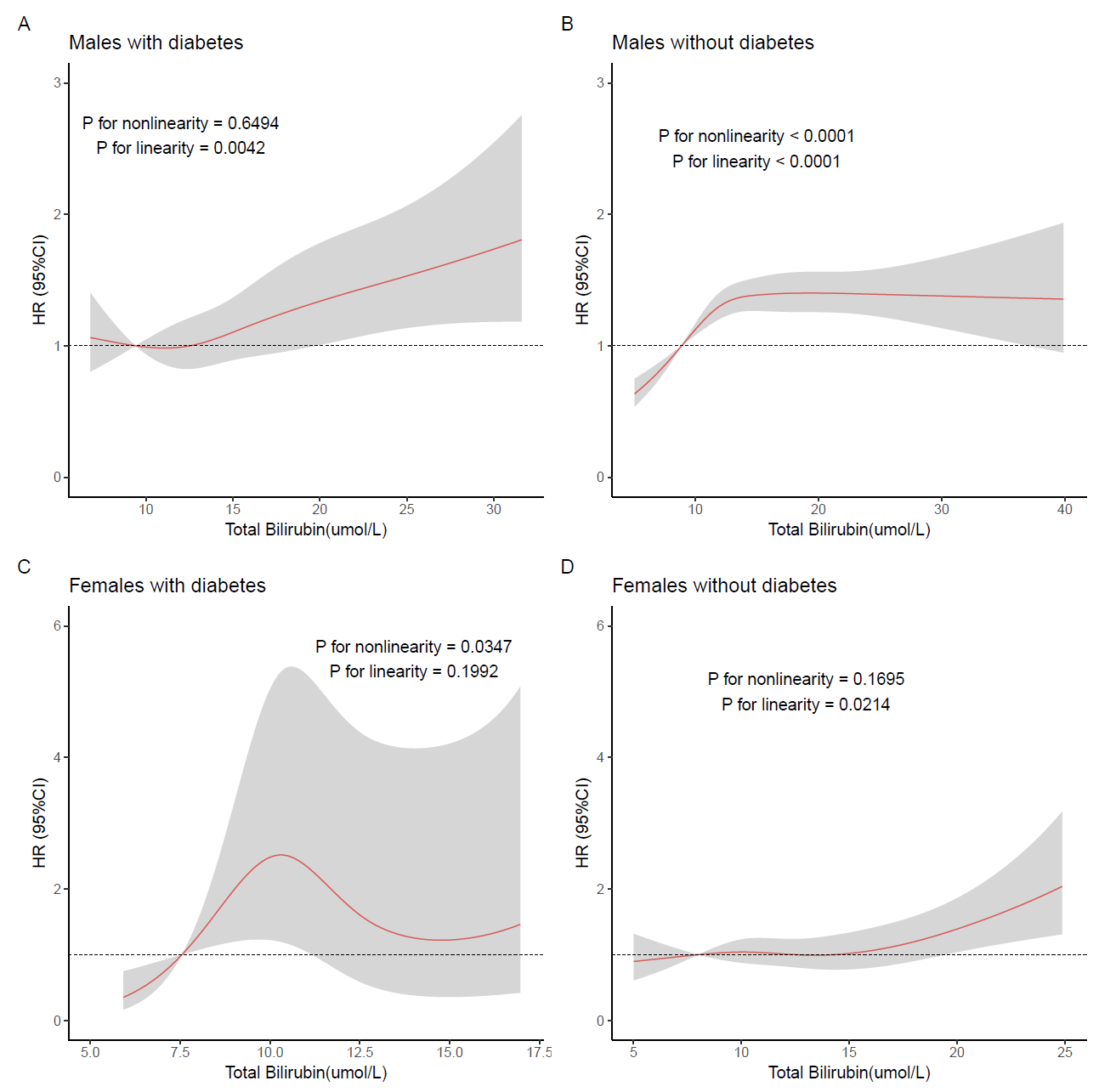
**

**Supplementary Figure S6** **Association of TBIL with the incidence of fundus arteriosclerosis in the diabetes subgroups.** A: Restricted cubic spline plot in male subgroup with diabetes; B: Restricted cubic spline plot in male subgroup without diabetes. C: Restricted cubic spline plot in female subgroup with diabetes; D: Restricted cubic spline plot in female subgroup without diabetes. According to restricted cubic spline regressions using four knots in males and females (percentiles 5, 35, 65 and 95), with the reference point set at percentile 12.5. Hazards ratios were adjusted for age, BMI, FBG, smoke, drunk, AST, ALT, γ-GT, TG, TP, Albumin, Globulin, CHOL, HDL, LDL, UA, BUN, hypertension status, diabetes status and fatty liver status, respectively.

HR: hazard ratio; CI: confidence interval.

**Supplementary Table S1 Baseline characteristics of participants in total populations**

| Characteristics  (Median [IQR]) | Total Bilirubin, μmol/L | | | | *P* value | *P* for trend |
| --- | --- | --- | --- | --- | --- | --- |
|  | Q1: ≤10.1  *n*=7058 | Q2: 10.1-12.7  *n*=6850 | Q3: 12.7-15.9  *n*=6821 | Q4: >15.9  *n*=6748 |  |  |
| Age, year | 47.00 [39.00, 54.00] | 48.00 [40.00, 55.00] | 48.00 [40.00, 55.00] | 47.00 [39.00, 54.00] | 0.003 | 0.702 |
| BMI, kg/m^2^ | 23.39 [21.36, 25.39] | 23.59 [21.45, 25.61] | 23.53 [21.35, 25.69] | 23.59 [21.36, 25.65] | 0.025 | <0.001 |
| ALT, U/L | 18.55 [13.00, 28.00] | 20.00 [14.00, 30.00] | 20.00 [14.00, 31.00] | 21.00 [15.00, 33.00] | <0.001 | <0.001 |
| AST, U/L | 19.00 [16.00, 24.00] | 20.00 [17.00, 25.00] | 21.00 [17.00, 25.00] | 21.00 [18.00, 26.00] | <0.001 | <0.001 |
| γ-GT, U/L | 20.00 [13.00, 34.00] | 22.00 [14.00, 37.00] | 22.00 [14.00, 39.00] | 24.00 [15.00, 42.00] | <0.001 | <0.001 |
| TP, g/L | 74.45 [71.93, 77.20] | 74.63 [72.10, 77.30] | 74.67 [72.20, 77.37] | 74.96 [72.44, 77.65] | <0.001 | 0.051 |
| Albumin, g/L | 46.20 [44.59, 47.84] | 46.58 [44.97, 48.18] | 46.81 [45.20, 48.40] | 47.20 [45.59, 48.84] | <0.001 | <0.001 |
| Globulin, g/L | 28.10 [25.98, 30.34] | 27.86 [25.87, 30.06] | 27.72 [25.77, 29.92] | 27.54 [25.67, 29.78] | <0.001 | <0.001 |
| TG, mmol/L | 1.27 [0.86, 1.94] | 1.27 [0.87, 1.89] | 1.24 [0.85, 1.86] | 1.24 [0.84, 1.88] | 0.005 | 0.126 |
| CHOL, mmol/L | 4.52 [4.00, 5.10] | 4.61 [4.08, 5.19] | 4.62 [4.10, 5.19] | 4.57 [4.07, 5.13] | <0.001 | 0.687 |
| HDL, mmol/L | 1.24 [1.05, 1.47] | 1.28 [1.09, 1.52] | 1.29 [1.10, 1.53] | 1.31 [1.11, 1.54] | <0.001 | <0.001 |
| LDL, mmol/L | 2.69 [2.25, 3.19] | 2.77 [2.30, 3.27] | 2.77 [2.32, 3.28] | 2.73 [2.29, 3.22] | <0.001 | 0.789 |
| FBG, mmol/L | 5.25 [4.98, 5.55] | 5.25 [4.99, 5.57] | 5.26 [4.98, 5.58] | 5.27 [4.99, 5.64] | <0.001 | 0.020 |
| UA, μmol/L | 323.2[263.8,382.8] | 332.2[272.9,394.0] | 337.3[277.2,396.9] | 348.8[292.1,402.9] | <0.001 | <0.001 |
| BUN, mmol/L | 5.04 [4.34, 5.85] | 4.99 [4.31, 5.76] | 4.95 [4.28, 5.69] | 4.91 [4.25, 5.67] | <0.001 | 0.005 |
| Smoke, *n* (%) |  |  |  |  |  |  |
| no | 1960 (42.9) | 2114 (44.5) | 2227 (43.8) | 2359 (43.1) | 0.396 | 0.897 |
| yes | 2606 (57.1) | 2639 (55.5) | 2860 (56.2) | 3116 (56.9) |  |  |
| Drunk, *n* (%) |  |  |  |  |  |  |
| no | 1814 (39.7) | 1675 (35.2) | 1632 (32.0) | 1576 (28.8) | <0.001 | <0.001 |
| yes | 2757 (60.3) | 3088 (64.8) | 3462 (68.0) | 3899 (71.2) |  |  |
| Hypertension, *n* (%) | |  |  |  |  |  |
| no | 6201 (87.9) | 5938 (86.7) | 5821 (85.3) | 5704 (84.5) | <0.001 | <0.001 |
| yes | 857 (12.1) | 912 (13.3) | 1000 (14.7) | 1044 (15.5) |  |  |
| Diabetes, *n* (%) |  |  |  |  |  |  |
| no | 6846 (97.0) | 6608 (96.5) | 6549 (96.0) | 6440 (95.4) | <0.001 | <0.001 |
| yes | 212 (3.0) | 242 (3.5) | 272 (4.0) | 308 (4.6) |  |  |
| Fatty liver, *n* (%) |  |  |  |  |  |  |
| no | 4758 (67.4) | 4423 (64.6) | 4484 (65.7) | 4450 (65.9) | 0.005 | 0.249 |
| yes | 2300 (32.6) | 2427 (35.4) | 2337 (34.3) | 2298 (34.1) |  |  |
| Fundus atherosclerosis, *n* (%) | |  |  |  |  |  |
| no | 6211 (88.0) | 5840 (85.3) | 5747 (84.3) | 5680 (84.2) | <0.001 | <0.001 |
| yes | 847 (12.0) | 1010 (14.7) | 1074 (15.7) | 1068 (15.8) |  |  |
| Gender, *n* (%) |  |  |  |  |  |  |
| male | 3801 (53.9) | 4193 (61.2) | 4606 (67.5) | 5136 (76.1) | <0.001 | <0.001 |
| female | 3257 (46.1) | 2657 (38.8) | 2215 (32.5) | 1612 (23.9) |  |  |

Abbreviations: ALT, alanine aminotransferase; AST, aspartate aminotransferase; γ-GT, γ-glutamyl transpeptidase; TP, serum total protein; TG, triacylglycerol; CHOL, cholesterol; HDL, high density lipoprotein; LDL, low Density Lipoprotein; FBG, fasting blood glucose; UA, uric acid; BUN, blood urea nitrogen; IQR: interquartile range.

**Supplementary Table S2 Cox regression analysis of TBIL and fundus arteriosclerosis in different populations in age subgroups**

| Subgroup | Level | Model 1^*^ | | Model 2^†^ | | Model 3^‡^ | |
| --- | --- | --- | --- | --- | --- | --- | --- |
|  |  | HR (95%CI) | ***P value*** | HR (95%CI) | ***P value*** | HR (95%CI) | ***P value*** |
| Males, Total Bilirubin (μmol/L, Median[IQR]) | | |  |  |  |  |  |
| Age, year |  |  |  |  |  |  |  |
| ≤60 | Q1 (9.0[≤10.6]) | 1(Reference) |  | 1(Reference) |  | 1(Reference) |  |
|  | Q2 (12.1[10.6-13.3]) | 1.194(1.061-1.344) | 0.003 | 1.215(1.077-1.370) | 0.002 | 1.201(1.063-1.355) | 0.003 |
|  | Q3 (14.8[13.3-16.7]) | 1.316(1.173-1.477) | <0.001 | 1.296(1.152-1.458) | <0.001 | 1.243(1.103-1.401) | <0.001 |
|  | Q4 (19.8[>16.7]) | 1.273(1.133-1.431) | <0.001 | 1.342(1.191-1.512) | <0.001 | 1.326(1.173-1.499) | <0.001 |
| ***P*** for trend |  | <0.001 |  | <0.001 |  | <0.001 |  |
| >60 | Q1 (8.9[≤10.3]) | 1(Reference) |  | 1(Reference) |  | 1(Reference) |  |
|  | Q2 (11.7[10.3-12.9]) | 1.593(1.277-1.987) | <0.001 | 1.545(1.234-1.934) | <0.001 | 1.515(1.207-1.902) | <0.001 |
|  | Q3 (14.5[12.9-16.3]) | 1.586(1.270-1.980) | <0.001 | 1.553(1.241-1.943) | <0.001 | 1.482(1.180-1.861) | 0.001 |
|  | Q4 (19.1[>16.3]) | 1.799(1.445-2.239) | <0.001 | 1.791(1.437-2.233) | <0.001 | 1.771(1.408-2.227) | <0.001 |
| ***P*** for trend |  | <0.001 |  | <0.001 |  | <0.001 |  |
| Females, Total Bilirubin (μmol/L, Median[IQR]) | | |  |  |  |  |  |
| Age, year |  |  |  |  |  |  |  |
| ≤60 | Q1 (8.0[≤9.4]) | 1(Reference) |  | 1(Reference) |  | 1(Reference) |  |
|  | Q2 (10.6[9.4-11.7]) | 1.018(0.821-1.262) | 0.871 | 1.102(0.800-1.518) | 0.554 | 1.053(0.760-1.461) | 0.755 |
|  | Q3 (13.0[11.7-14.5]) | 0.903(0.724-1.125) | 0.362 | 0.936(0.671-1.306) | 0.699 | 1.053(0.750-1.489) | 0.767 |
|  | Q4 (17.2[>14.5]) | 0.989(0.796-1.228) | 0.917 | 1.296(0.945-1.776) | 0.108 | 1.467(1.058-2.033) | 0.022 |
| ***P*** for trend |  | 0.748 |  | 0.151 |  | 0.018 |  |
| >60 | Q1 (8.0[≤9.2]) | 1(Reference) |  | 1(Reference) |  | 1(Reference) |  |
|  | Q2 (10.56[9.2-11.6]) | 1.041(0.746-1.453) | 0.814 | 1.147(0.695-1.893) | 0.591 | 1.319(0.755-2.304) | 0.330 |
|  | Q3 (12.6[11.6-13.8]) | 1.255(0.907-1.737) | 0.171 | 1.433(0.865-2.373) | 0.162 | 1.378(0.805-2.360) | 0.243 |
|  | Q4 (15.9[>13.8]) | 1.046(0.749-1.460) | 0.794 | 0.778(0.458-1.317) | 0.349 | 0.866(0.485-1.547) | 0.627 |
| ***P*** for trend |  | 0.634 |  | 0.422 |  | 0.622 |  |

In the age subgroups, univariate and multivariate Cox regression analysis of the relationship between total bilirubin and fundus arteriosclerosis, in males and females. ^*^: Model1, non-adjusted; ^†^: Model2, adjusted for age, BMI, FBG, smoke, drunk; ^‡^: Model3, adjusted for age, BMI, FBG, smoke, drunk, AST, ALT, γ-GT, TG, TP, Albumin, Globulin, CHOL, HDL, LDL, UA, BUN, hypertension status, diabetes status and fatty liver status.

HR: hazard ratio; CI: confidence interval; IQR: interquartile range.

**Supplementary Table S3 Cox regression analysis of TBIL and fundus arteriosclerosis in different populations in BMI subgroups**

| Subgroup | Level | Model 1^*^ | | Model 2^†^ | | Model 3^‡^ | |
| --- | --- | --- | --- | --- | --- | --- | --- |
|  |  | HR (95%CI) | ***P value*** | HR (95%CI) | ***P value*** | HR (95%CI) | ***P value*** |
| Males, Total Bilirubin (μmol/L, Median[IQR]) | | |  |  |  |  |  |
| BMI, kg/m^2^ |  |  |  |  |  |  |  |
| ≤25 | Q1 (9.0[≤10.6]) | 1(Reference) |  | 1(Reference) |  | 1(Reference) |  |
|  | Q2 (12.1[10.6-13.3]) | 1.167(1.002-1.359) | 0.048 | 1.151(0.985-1.344) | 0.761 | 1.143(0.977-1.337) | 0.094 |
|  | Q3 (14.9[13.3-16.8]) | 1.151(0.988-1.340) | 0.071 | 1.274(1.091-1.488) | 0.002 | 1.233(1.053-1.444) | 0.009 |
|  | Q4 (20.0[>16.8]) | 1.177(1.011-1.372) | 0.036 | 1.335(1.143-1.559) | <0.001 | 1.320(1.124-1.551) | 0.001 |
| ***P*** for trend |  | 0.069 |  | <0.001 |  | <0.001 |  |
| >25 | Q1 (9.0[≤10.6]) | 1(Reference) |  | 1(Reference) |  | 1(Reference) |  |
|  | Q2 (12.0[10.6-13.2]) | 1.329(1.154-1.531) | <0.001 | 1.299(1.125-1.500) | <0.001 | 1.330(1.150-1.537) | <0.001 |
|  | Q3 (14.7[13.2-16.4]) | 1.435(1.250-1.648) | <0.001 | 1.414(1.229-1.626) | <0.001 | 1.376(1.194-1.586) | <0.001 |
|  | Q4 (19.7[>16.4]) | 1.470(1.281-1.687) | <0.001 | 1.411(1.226-1.623) | <0.001 | 1.402(1.213-1.621) | <0.001 |
| ***P*** for trend |  | <0.001 |  | <0.001 |  | <0.001 |  |
| Females, Total Bilirubin (μmol/L, Median[IQR]) | | |  |  |  |  |  |
| BMI, kg/m^2^ |  |  |  |  |  |  |  |
| ≤25 | Q1 (8.0[≤9.4]) | 1(Reference) |  | 1(Reference) |  | 1(Reference) |  |
|  | Q2 (10.6[9.4-11.8]) | 1.007(0.807-1.255) | 0.954 | 0.902(0.646-1.261) | 0.547 | 0.867(0.617-1.225) | 0.423 |
|  | Q3 (13.1[11.8-14.5]) | 0.944(0.752-1.184) | 0.616 | 0.951(0.669-1.352) | 0.778 | 0.984(0.683-1.417) | 0.929 |
|  | Q4 (17.2[>14.5]) | 0.935(0.746-1.173) | 0.563 | 1.153(0.820-1.621) | 0.412 | 1.251(0.872-1.794) | 0.224 |
| ***P*** for trend |  | 0.478 |  | 0.320 |  | 0.133 |  |
| >25 | Q1 (7.7[≤9.1]) | 1(Reference) |  | 1(Reference) |  | 1(Reference) |  |
|  | Q2 (10.2[9.1-11.3]) | 1.268(0.921-1.745) | 0.145 | 1.170(0.733-1.869) | 0.511 | 1.015(0.623-1.652) | 0.954 |
|  | Q3 (12.5[11.3-13.9]) | 1.374(1.004-1.880) | 0.147 | 1.028(0.639-1.652) | 0.910 | 0.948(0.574-1.565) | 0.833 |
|  | Q4 (16.4[>13.9]) | 1.242(0.900-1.714) | 0.187 | 1.080(0.678-1.721) | 0.745 | 1.038(0.636-1.696) | 0.880 |
| ***P*** for trend |  | 0.219 |  | 0.942 |  | 0.906 |  |

In the BMI subgroups, univariate and multivariate Cox regression analysis of the relationship between total bilirubin and fundus arteriosclerosis, in males and females. ^*^: Model1, non-adjusted; ^†^: Model2, adjusted for age, BMI, FBG, smoke, drunk; ^‡^: Model3, adjusted for age, BMI, FBG, smoke, drunk, AST, ALT, γ-GT, TG, TP, Albumin, Globulin, CHOL, HDL, LDL, UA, BUN, hypertension status, diabetes status and fatty liver status.

HR: hazard ratio; CI: confidence interval; IQR: interquartile range.

**Supplementary Table S4 Cox regression analysis of TBIL and fundus arteriosclerosis in different populations in hypertension subgroups**

| Subgroup | Level | Model 1^*^ | | Model 2^†^ | | Model 3^‡^ | |
| --- | --- | --- | --- | --- | --- | --- | --- |
|  |  | HR (95%CI) | ***P value*** | HR (95%CI) | ***P value*** | HR (95%CI) | ***P value*** |
| Males, Total Bilirubin (μmol/L, Median[IQR]) | | |  |  |  |  |  |
| Hypertension |  |  |  |  |  |  |  |
| yes | Q1 (9.28[≤10.8]) | 1(Reference) |  | 1(Reference) |  | 1(Reference) |  |
|  | Q2 (12.38[10.8-13.6]) | 1.176(1.005-1.376) | 0.043 | 1.162(0.991-1.365) | 0.066 | 1.135(0.965-1.335) | 0.126 |
|  | Q3 (15.1[13.6-16.9]) | 1.136(0.970-1.330) | 0.114 | 1.148(0.977-1.349) | 0.093 | 1.099(0.932-1.295) | 0.261 |
|  | Q4 (19.95[>16.9]) | 1.239(1.061-1.447) | 0.007 | 1.273(1.087-1.491) | 0.003 | 1.253(1.063-1.475) | 0.007 |
| ***P*** for trend |  | 0.015 |  | 0.005 |  | 0.012 |  |
| no | Q1 (8.9[≤10.5]) | 1(Reference) |  | 1(Reference) |  | 1(Reference) |  |
|  | Q2 (12.0[10.5-13.2]) | 1.245(1.088-1.424) | 0.001 | 1.328(1.158-1.523) | <0.001 | 1.371(1.195-1.574) | <0.001 |
|  | Q3 (14.8[13.2-16.6]) | 1.294(1.132-1.479) | <0.001 | 1.408(1.229-1.613) | <0.001 | 1.424(1.241-1.633) | <0.001 |
|  | Q4 (19.6[>16.6]) | 1.231(1.075-1.410) | 0.003 | 1.453(1.266-1.668) | <0.001 | 1.545(1.342-1.779) | <0.001 |
| ***P*** for trend |  | 0.007 |  | <0.001 |  | <0.001 |  |
| Females, Total Bilirubin (μmol/L, Median[IQR]) | | |  |  |  |  |  |
| Hypertension |  |  |  |  |  |  |  |
| yes | Q1 (8.0[≤9.4]) | 1(Reference) |  | 1(Reference) |  | 1(Reference) |  |
|  | Q2 (10.6[9.4-11.5]) | 1.225(0.884-1.698) | 0.223 | 1.548(0.943-2.543) | 0.084 | 1.419(0.835-2.410) | 0.196 |
|  | Q3 (12.6[11.5-14.2]) | 1.241(0.892-1.727) | 0.201 | 1.137(0.666-1.941) | 0.637 | 0.844(0.470-1.515) | 0.571 |
|  | Q4 (16.5[>14.2]) | 1.332(0.965-1.840) | 0.081 | 1.604(0.957-2.688) | 0.073 | 1.616(0.919-2.842) | 0.096 |
| ***P*** for trend |  | 0.100 |  | 0.163 |  | 0.219 |  |
| no | Q1 (8.0[≤9.4]) | 1(Reference) |  | 1(Reference) |  | 1(Reference) |  |
|  | Q2 (10.6[9.4-11.7]) | 0.919(0.739-1.143) | 0.449 | 0.837(0.605-1.157) | 0.281 | 0.868(0.626-1.205) | 0.400 |
|  | Q3 (13.0[11.7-14.4]) | 0.955(0.770-1.184) | 0.677 | 0.815(0.588-1.129) | 0.219 | 0.930(0.667-1.296) | 0.667 |
|  | Q4 (17.1[>14.4]) | 0.827(0.661-1.033) | 0.094 | 0.973(0.710-1.332) | 0.862 | 1.088(0.782-1.515) | 0.616 |
| ***P*** for trend |  | 0.121 |  | 0.999 |  | 0.469 |  |

In the hypertension subgroups, univariate and multivariate Cox regression analysis of the relationship between total bilirubin and fundus arteriosclerosis, in males and females. ^*^: Model1, non-adjusted; ^†^: Model2, adjusted for age, BMI, FBG, smoke, drunk; ^‡^: Model3, adjusted for age, BMI, FBG, smoke, drunk, AST, ALT, γ-GT, TG, TP, Albumin, Globulin, CHOL, HDL, LDL, UA, BUN, hypertension status, diabetes status and fatty liver status.

HR: hazard ratio; CI: confidence interval; IQR: interquartile range.

**Supplementary Table S5 Cox regression analysis of TBIL and fundus arteriosclerosis in different populations in diabetes subgroups**

| Subgroup | Level | Model 1^*^ | | Model 2^†^ | | Model 3^‡^ | |
| --- | --- | --- | --- | --- | --- | --- | --- |
|  |  | HR (95%CI) | ***P value*** | HR (95%CI) | ***P value*** | HR (95%CI) | ***P value*** |
| Males, Total Bilirubin (μmol/L, Median[IQR]) | | |  |  |  |  |  |
| Diabetes |  |  |  |  |  |  |  |
| yes | Q1 (9.28[≤10.8]) | 1(Reference) |  | 1(Reference) |  | 1(Reference) |  |
|  | Q2 (12.38[10.8-13.6]) | 1.058(0.795-1.409) | 0.697 | 0.990(0.740-1.325) | 0.946 | 0.955(0.711-1.283) | 0.760 |
|  | Q3 (15.1[13.6-16.9]) | 1.190(0.901-1.572) | 0.220 | 1.066(0.804-1.414) | 0.656 | 1.124(0.840-1.504) | 0.431 |
|  | Q4 (19.95[>16.9]) | 1.234(0.936-1.628) | 0.136 | 1.259(0.951-1.667) | 0.107 | 1.356(1.011-1.820) | 0.042 |
| ***P*** for trend |  | 0.103 |  | 0.075 |  | 0.020 |  |
| no | Q1 (9.0[≤10.6]) | 1(Reference) |  | 1(Reference) |  | 1(Reference) |  |
|  | Q2 (12.0[10.6-13.2]) | 1.279(1.145-1.428) | <0.001 | 1.289(1.152-1.442) | <0.001 | 1.284(1.147-1.439) | <0.001 |
|  | Q3 (14.7[13.2-16.6]) | 1.308(1.173-1.459) | <0.001 | 1.412(1.263-1.577) | <0.001 | 1.372(1.226-1.535) | <0.001 |
|  | Q4 (19.7[>16.6]) | 1.271(1.139-1.419) | <0.001 | 1.420(1.269-1.588) | <0.001 | 1.404(1.251-1.576) | <0.001 |
| ***P*** for trend |  | <0.001 |  | <0.001 |  | <0.001 |  |
| Females, Total Bilirubin (μmol/L, Median[IQR]) | | |  |  |  |  |  |
| Diabetes |  |  |  |  |  |  |  |
| yes | Q1 (7.55[≤9.15]) | 1(Reference) |  | 1(Reference) |  | 1(Reference) |  |
|  | Q2 (10.75[9.15-11.4]) | 1.816(0.903-3.653) | 0.094 | 2.812(1.058-7.524) | 0.038 | 2.552(0.836-7.796) | 0.100 |
|  | Q3 (12.5[11.4-13.85]) | 2.138(1.076-4.247) | 0.030 | 2.826(1.029-7.761) | 0.044 | 1.967(0.623-6.204) | 0.249 |
|  | Q4 (15.95[>13.85]) | 1.427(0.686-2.967) | 0.341 | 1.822(0.685-4.841) | 0.229 | 1.472(0.420-5.154) | 0.546 |
| ***P*** for trend |  | 0.325 |  | 0.318 |  | 0.631 |  |
| no | Q1 (8.0[≤9.4]) | 1(Reference) |  | 1(Reference) |  | 1(Reference) |  |
|  | Q2 (10.6[9.4-11.7]) | 0989(0.821-1.193) | 0.911 | 0.900(0.678-1.193) | 0.462 | 0.937(0.705-1.246) | 0.655 |
|  | Q3 (13.0[11.7-14.4]) | 0.967(0.812-1.167) | 0.728 | 0.812(0.606-1.089) | 0.164 | 0.867(0.643-1.169) | 0.350 |
|  | Q4 (17.1[>14.4]) | 0.927(0.767-1.121) | 0.434 | 1.070(0.811-1.413) | 0.636 | 1.244(0.933-1.657) | 0.137 |
| ***P*** for trend |  | 0.407 |  | 0.602 |  | 0.130 |  |

In the diabetes subgroups, univariate and multivariate Cox regression analysis of the relationship between total bilirubin and fundus arteriosclerosis, in males and females. ^*^: Model1, non-adjusted; ^†^: Model2, adjusted for age, BMI, FBG, smoke, drunk; ^‡^: Model3, adjusted for age, BMI, FBG, smoke, drunk, AST, ALT, γ-GT, TG, TP, Albumin, Globulin, CHOL, HDL, LDL, UA, BUN, hypertension status, diabetes status and fatty liver status.

HR: hazard ratio; CI: confidence interval; IQR: interquartile range.

**Supplementary Table S6 Cox regression analysis of TBIL and fundus arteriosclerosis in Gilbert’s population**

| Level | Cases | Model 1^*^ | | Model 2^†^ | | Model 3^‡^ | | |
| --- | --- | --- | --- | --- | --- | --- | --- | --- |
|  |  | HR (95%CI) | ***P value*** | HR (95%CI) | ***P value*** | HR (95%CI) | ***P value*** |  |
| **Overall**, Total Bilirubin (μmol/L, Median[IQR]) | | |  |  |  |  |  | |
| Q1 (24.3[≤25.40]) | 77 | 1(Reference) |  | 1(Reference) |  | 1(Reference) |  | |
| Q2 (26.5[25.40-27.50]) | 37 | 0.467(0.316-0.691) | <0.001 | 0.683(0.452-1.032) | 0.070 | 0.861(0.551-1.347) | 0.512 | |
| Q3 (28.8[27.50-30.95]) | 40 | 0.511(0.349-0.749) | 0.001 | 0.871(0.583-1.299) | 0.498 | 1.003(0.653-1.541) | 0.989 | |
| Q4 (34.5[>30.95]) | 33 | 0.402(0.268-0.605) | <0.001 | 0.794(0.517-1.220) | 0.293 | 0.980(0.608-1.579) | 0.934 | |
| ***P* for *trend*** |  | <0.001 |  | 0.440 |  | 0.921 |  | |
| **Male**, Total Bilirubin (μmol/L, Median[IQR]) | | |  |  |  |  |  | |
| Q1 (24.3[≤25.40]) | 64 | 1(Reference) |  | 1(Reference) |  | 1(Reference) |  | |
| Q2 (26.5[25.40-27.50]) | 35 | 0.539(0.357-0.814) | 0.003 | 0.662(0.435-1.001) | 0.055 | 0.598(0.382-0.935) | 0.024 | |
| Q3 (29.1[27.50-31.20]) | 38 | 0.546(0.366-0.816) | 0.003 | 0.833(0.553-1.256) | 0.384 | 0.760(0.493-1.171) | 0.113 | |
| Q4 (34.8[>31.20]) | 29 | 0.424(0.273-0.657) | <0.001 | 0.768(0.492-1.200) | 0.246 | 0.682(0.418-0.911) | 0.003 | |
| ***P* for *trend*** |  | <0.001 |  | 0.379 |  | 0.263 |  | |
| **Female**, Total Bilirubin (μmol/L, Median[IQR]) | | |  |  |  |  |  | |
| Q1 (23.9[≤25.30]) | 13 | 1(Reference) |  | 1(Reference) |  | 1(Reference) |  | |
| Q2 (26.45[25.30-27.20]) | 1 | 0.069(0.009-0.529) | 0.010 | 1.092(0.108-11.030) | 0.941 | - | - | |
| Q3 (28.45[27.20-29.45]) | 4 | 0.308(0.100-0.945) | 0.040 | 2.713(0.316-23.285) | 0.363 | - | - | |
| Q4 (33.4[>29.45]) | 3 | 0.216(0.062-0.760) | 0.017 | 1.809(0.184-17.802) | 0.611 | - | - | |
| ***P* for *trend*** |  | 0.020 |  | 0.470 |  | - |  | |

HR and 95% CI for changes in TBIL for fundus arteriosclerosis incidence according to quartiles of TBIL in total population, male and female. Notes: in women, the sample size was too small to be calculated. ^*^: Model1, non-adjusted; ^†^: Model2, adjusted for age, BMI, FBG, smoke, drunk; ^‡^: Model3, adjusted for age, BMI, FBG, smoke, drunk, AST, ALT, γ-GT, TG, TP, Albumin, Globulin, CHOL, HDL, LDL, UA, BUN, hypertension status, diabetes status and fatty liver status.

HR: hazard ratio; CI: confidence interval; IQR: interquartile range.

**Supplementary Table S7 Cox regression analysis of TBIL and fundus arteriosclerosis in total population**

|  |  | Model 1^*^ | | Model 2^†^ | | Model 3^‡^ | |
| --- | --- | --- | --- | --- | --- | --- | --- |
|  |  | HR (95%CI) | ***P value*** | HR (95%CI) | ***P value*** | HR (95%CI) | ***P value*** |
| TBIL levels | Q1 | 1(Reference) |  | 1(Reference) |  | 1(Reference) |  |
|  | Q2 | 1.254(1.144-1.374) | <0.001 | 1.237(1.117-1.371) | <0.001 | 1.232(1.111-1.366) | <0.001 |
|  | Q3 | 1.348(1.232-1.475) | <0.001 | 1.337(1.210-1.478) | <0.001 | 1.312(1.184-1.453) | <0.001 |
|  | Q4 | 1.357(1.240-1.485) | <0.001 | 1.387(1.256-1.532) | <0.001 | 1.366(1.231-1.515) | <0.001 |
| female |  |  |  | 1.514(1.347-1.701) | <0.001 | 1.641(1.446-1.862) | <0.001 |
| age |  |  |  | 1.117(1.113-1.121) | <0.001 | 1.107(1.103-1.111) | <0.001 |
| BMI |  |  |  | 1.143(1.133-1.153) | <0.001 | 1.095(1.082-1.108) | <0.001 |
| FBG |  |  |  | 1.080(1.054-1.106) | <0.001 | 0.977(0.943-1.012) | 0.195 |
| Smoke |  |  |  | 0.956(0.887-1.030) | 0.234 | 0.900(0.833-0.971) | 0.007 |
| Drunk |  |  |  | 1.335(1.231-1.448) | <0.001 | 1.295(1.192-1.408) | <0.001 |
| AST |  |  |  |  |  | 1.001(0.996-1.003) | 0.681 |
| ALT |  |  |  |  |  | 1.001(0.998-1.003) | 0.124 |
| γ-GT |  |  |  |  |  | 0.999(0.998-1.000) | 0.076 |
| TG |  |  |  |  |  | 1.027(0.978-1.079) | 0.285 |
| TP |  |  |  |  |  | 1.193(1.115-1.278) | <0.001 |
| Albumin |  |  |  |  |  | 0.830(0.773-0.890) | <0.001 |
| Globulin |  |  |  |  |  | 0.807(0.753-0.864) | <0.001 |
| CHOL |  |  |  |  |  | 1.192(1.046-1.359) | 0.008 |
| HDL |  |  |  |  |  | 0.709(0.587-0.856) | <0.001 |
| LDL |  |  |  |  |  | 1.044(0.909-1.198) | 0.546 |
| UA |  |  |  |  |  | 1.001(1.000-1.001) | <0.001 |
| BUN |  |  |  |  |  | 1.012(0.993-1.032) | 0.228 |
| Hypertension |  |  |  |  |  | 2.057(1.909-2.216) | <0.001 |
| Diabetes |  |  |  |  |  | 1.380(1.201-1.586) | <0.001 |
| Fatty liver |  |  |  |  |  | 1.152(1.064-1.248) | 0.001 |

HR and 95% CI for changes in TBIL for fundus arteriosclerosis incidence according to quartiles of TBIL in total population, male and female. ^*^: Model1, non-adjusted; ^†^: Model2, adjusted for gender, age, BMI, FBG, smoke, drunk; ^‡^: Model3, adjusted for gender, age, BMI, FBG, smoke, drunk, AST, ALT, γ-GT, TG, TP, Albumin, Globulin, CHOL, HDL, LDL, UA, BUN, hypertension status, diabetes status and fatty liver status.

HR: hazard ratio; CI: confidence interval; IQR: interquartile range.

**Supplementary Table S8 Cox regression analysis of TBIL and fundus arteriosclerosis in males**

|  |  | Model 1^*^ | | Model 2^†^ | | Model 3^‡^ | |
| --- | --- | --- | --- | --- | --- | --- | --- |
|  |  | HR (95%CI) | ***P value*** | HR (95%CI) | ***P value*** | HR (95%CI) | ***P value*** |
| TBIL levels | Q1 | 1(Reference) |  | 1(Reference) |  | 1(Reference) |  |
|  | Q2 | 1.246(1.123-1.381) | <0.001 | 1.241(1.117-1.378) | <0.001 | 1.217(1.095-1.354) | <0.001 |
|  | Q3 | 1.310(1.182-1.451) | <0.001 | 1.321(1.190-1.465) | <0.001 | 1.255(1.128-1.396) | <0.001 |
|  | Q4 | 1.300(1.173-1.439) | <0.001 | 1.431(1.289-1.587) | <0.001 | 1.396(1.254-1.555) | <0.001 |
| age |  |  |  | 1.118(1.114-1.122) | <0.001 | 1.109(1.104-1.113) | <0.001 |
| BMI |  |  |  | 1.143(1.132-1.153) | <0.001 | 1.097(1.083-1.110) | <0.001 |
| FBG |  |  |  | 1.082(1.055-1.110) | <0.001 | 0.984(0.978-1.021) | 0.393 |
| Smoke |  |  |  | 0.946(0.877-1.020) | 0.147 | 0.896(0.829-0.969) | 0.006 |
| Drunk |  |  |  | 1.354(1.243-1.474) | <0.001 | 1.299(1.190-1.417) | <0.001 |
| AST |  |  |  |  |  | 1.000(0.998-1.003) | 0.924 |
| ALT |  |  |  |  |  | 1.001(0.999-1.003) | 0.124 |
| γ-GT |  |  |  |  |  | 0.999(0.998-1.000) | 0.030 |
| TG |  |  |  |  |  | 1.034(0.983-1.089) | 0.197 |
| TP |  |  |  |  |  | 1.190(1.110-1.276) | <0.001 |
| Albumin |  |  |  |  |  | 0.830(0.773-0.892) | <0.001 |
| Globulin |  |  |  |  |  | 0.807(0.752-0.866) | <0.001 |
| CHOL |  |  |  |  |  | 1.184(1.032-1.358) | 0.016 |
| HDL |  |  |  |  |  | 0.710(0.579-0.870) | 0.001 |
| LDL |  |  |  |  |  | 1.050(0.908-1.214) | 0.512 |
| UA |  |  |  |  |  | 1.001(1.000-1.001) | 0.001 |
| BUN |  |  |  |  |  | 1.005(0.982-1.028) | 0.673 |
| Hypertension |  |  |  |  |  | 2.139(1.977-2.315) | <0.001 |
| Diabetes |  |  |  |  |  | 1.405(1.212-1.629) | <0.001 |
| Fatty liver |  |  |  |  |  | 1.135(1.042-1.235) | 0.004 |

HR and 95% CI for changes in TBIL for fundus arteriosclerosis incidence according to quartiles of TBIL in total population, male and female. ^*^: Model1, non-adjusted; ^†^: Model2, adjusted for age, BMI, FBG, smoke, drunk; ^‡^: Model3, adjusted for age, BMI, FBG, smoke, drunk, AST, ALT, γ-GT, TG, TP, Albumin, Globulin, CHOL, HDL, LDL, UA, BUN, hypertension status, diabetes status and fatty liver status.

HR: hazard ratio; CI: confidence interval; IQR: interquartile range.

**Supplementary Table S9 Cox regression analysis of TBIL and fundus arteriosclerosis in females**

|  |  | Model 1^*^ | | Model 2^†^ | | Model 3^‡^ | |
| --- | --- | --- | --- | --- | --- | --- | --- |
|  |  | HR (95%CI) | ***P value*** | HR (95%CI) | ***P value*** | HR (95%CI) | ***P value*** |
| TBIL levels | Q1 | 1(Reference) |  | 1(Reference) |  | 1(Reference) |  |
|  | Q2 | 1.031(0.861-1.234) | 0.742 | 1.063(0.812-1.392) | 0.655 | 1.054(0.800-1.389) | 0.707 |
|  | Q3 | 1.010(0.843-1.210) | 0.914 | 0.970(0.724-1.281) | 0.828 | 1.001(0.757-1.344) | 0.954 |
|  | Q4 | 0.934(0.777-1.122) | 0.463 | 1.193(0.913-1.560) | 0.196 | 1.308(0.987-1.734) | 0.062 |
| age |  |  |  | 1.112(1.102-1.123) | <0.001 | 1.090(1.078-1.103) | <0.001 |
| BMI |  |  |  | 1.163(1.128-1.199) | <0.001 | 1.092(1.050-1.134) | <0.001 |
| FBG |  |  |  | 1.041(0.956-1.133) | 0.354 | 0.935(0.826-1.058) | 0.288 |
| Smoke |  |  |  | 1.312(0.864-1.994) | 0.203 | 1.268(0.793-2.027) | 0.322 |
| Drunk |  |  |  | 1.079(0.806-1.444) | 0.609 | 1.047(0.775-1.414) | 0.767 |
| AST |  |  |  |  |  | 1.032(1.012-1.053) | 0.002 |
| ALT |  |  |  |  |  | 0.989(0.977-1.002) | 0.093 |
| γ-GT |  |  |  |  |  | 1.002(0.998-1.005) | 0.332 |
| TG |  |  |  |  |  | 1.028(0.863-1.226) | 0.756 |
| TP |  |  |  |  |  | 1.417(0.791-2.541) | 0.242 |
| Albumin |  |  |  |  |  | 0.695(0.387-1.249) | 0.224 |
| Globulin |  |  |  |  |  | 0.678(0.378-1.216) | 0.193 |
| CHOL |  |  |  |  |  | 1.114(0.728-1.706) | 0.619 |
| HDL |  |  |  |  |  | 0.762(0.441-1.314) | 0.328 |
| LDL |  |  |  |  |  | 1.220(0.782-1.903) | 0.381 |
| UA |  |  |  |  |  | 1.002(1.000-1.003) | 0.030 |
| BUN |  |  |  |  |  | 1.085(1.012-1.163) | 0.022 |
| Hypertension |  |  |  |  |  | 1.453(1.149-1.838) | 0.002 |
| Diabetes |  |  |  |  |  | 1.016(0.641-1.608) | 0.948 |
| Fatty liver |  |  |  |  |  | 1.436(1.134-1.820) | 0.003 |

HR and 95% CI for changes in TBIL for fundus arteriosclerosis incidence according to quartiles of TBIL in total population, male and female. ^*^: Model1, non-adjusted; ^†^: Model2, adjusted for age, BMI, FBG, smoke, drunk; ^‡^: Model3, adjusted for age, BMI, FBG, smoke, drunk, AST, ALT, γ-GT, TG, TP, Albumin, Globulin, CHOL, HDL, LDL, UA, BUN, hypertension status, diabetes status and fatty liver status.

HR: hazard ratio; CI: confidence interval; IQR: interquartile range.

**Supplementary Table S10 Cox regression analysis of DBIL and fundus arteriosclerosis in different populations**

| Level | Cases | Model 1^*^ | | Model 2^†^ | | Model 3^‡^ | |
| --- | --- | --- | --- | --- | --- | --- | --- |
|  |  | HR (95%CI) | ***P value*** | HR (95%CI) | ***P value*** | HR (95%CI) | ***P value*** |
| **Overall**, Direct Bilirubin (μmol/L, Median [IQR]) | | |  |  |  |  |  |
| Q1 (2.5[≤3.0]) | 1283 | 1(Reference) |  | 1(Reference) |  | 1(Reference) |  |
| Q2 (3.5[3.0-3.9]) | 1075 | 0.798(0.736-0.865) | <0.001 | 0.900(0.822-0.985) | 0.022 | 0.932(0.850-1.022) | 0.132 |
| Q3 (4.4[3.9-5.0]) | 915 | 0.726(0.667-0.791) | <0.001 | 0.856(0.780-0.940) | 0.001 | 0.903(0.819-0.996) | 0.042 |
| Q4 (6.1[>5.0]) | 726 | 0.576(0.526-0.631) | <0.001 | 0.704(0.638-0.778) | <0.001 | 0.771(0.693-0.859) | <0.001 |
| ***P* for *trend*** |  | <0.001 |  | <0.001 |  | <0.001 |  |
| **Male**, Total Bilirubin (μmol/L, Median [IQR]) | | |  |  |  |  |  |
| Q1 (2.7[≤3.2]) | 976 | 1(Reference) |  | 1(Reference) |  | 1(Reference) |  |
| Q2 (3.7[3.2-4.1]) | 835 | 0.865(0.788-0.948) | 0.002 | 0.944(0.859-1.036) | 0.225 | 0.962(0.875-1.059) | 0.433 |
| Q3 (4.7[4.1-5.3]) | 745 | 0.727(0.661-0.799) | <0.001 | 0.857(0.778-0.944) | 0.002 | 0.896(0.810-0.991) | 0.033 |
| Q4 (6.4[>5.3]) | 520 | 0.537(0.483-0.597) | <0.001 | 0.708(0.636-0.789) | <0.001 | 0.761(0.677-0.854) | <0.001 |
| ***P* for *trend*** |  | <0.001 |  | <0.001 |  | <0.001 |  |
| **Female**, Total Bilirubin (μmol/L, Median [IQR]) | | |  |  |  |  |  |
| Q1 (2.3[≤2.7]) | 390 | 1(Reference) |  | 1(Reference) |  | 1(Reference) |  |
| Q2 (3.1[2.7-3.5]) | 261 | 0.610(0.521-0.713) | <0.001 | 0.717(0.569-0.904) | 0.005 | 0.751(0.590-0.955) | 0.019 |
| Q3 (3.9[3.5-4.4]) | 151 | 0.397(0.329-0.479) | <0.001 | 0.555(0.420-0.734) | <0.001 | 0.599(0.447-0.804) | 0.001 |
| Q4 (5.3[>4.4]) | 121 | 0.300(0.245-0.368) | <0.001 | 0.537(0.400-0.720) | <0.001 | 0.653(0.474-0.900) | 0.009 |
| ***P* for *trend*** |  | <0.001 |  | <0.001 |  | 0.002 |  |

HR and 95% CI for changes in DBIL for fundus arteriosclerosis incidence according to quartiles of TBIL in total population, male and female. ^*^: Model1, non-adjusted; ^†^: Model2, adjusted for age, BMI, FBG, smoke, drunk; ^‡^: Model3, adjusted for age, BMI, FBG, smoke, drunk, AST, ALT, γ-GT, TG, TP, Albumin, Globulin, CHOL, HDL, LDL, UA, BUN, hypertension status, diabetes status and fatty liver status.

HR: hazard ratio; CI: confidence interval; IQR: interquartile range.

**Supplementary Table S11 Cox regression analysis of IBIL and fundus arteriosclerosis in different populations**

| Level | Cases | Model 1^*^ | | Model 2^†^ | | Model 3^‡^ | |
| --- | --- | --- | --- | --- | --- | --- | --- |
|  |  | HR (95%CI) | ***P value*** | HR (95%CI) | ***P value*** | HR (95%CI) | ***P value*** |
| **Overall**, Direct Bilirubin (μmol/L, Median [IQR]) | | |  |  |  |  |  |
| Q1 (2.5[≤6.8]) | 693 | 1(Reference) |  | 1(Reference) |  | 1(Reference) |  |
| Q2 (3.5[6.8-8.8]) | 946 | 1.378(1.250-1.520) | <0.001 | 1.357(1.217-1.513) | <0.001 | 1.340(1.200-1.496) | <0.001 |
| Q3 (4.4[8.8-11.1]) | 1087 | 1.721(1.564-1.893) | <0.001 | 1.592(1.432-1.769) | <0.001 | 1.509(1.355-1.681) | <0.001 |
| Q4 (6.1[>11.1]) | 1273 | 1.986(1.810-2.178) | <0.001 | 1.822(1.645-2.019) | <0.001 | 1.742(1.566-1.937) | <0.001 |
| ***P* for *trend*** |  | <0.001 |  | <0.001 |  | <0.001 |  |
| **Male**, Total Bilirubin (μmol/L, Median [IQR]) | | |  |  |  |  |  |
| Q1 (5.9[≤7.1]) | 550 | 1(Reference) |  | 1(Reference) |  | 1(Reference) |  |
| Q2 (8.2[7.1-9.1]) | 735 | 1.417(1.269-1.582) | <0.001 | 1.484(1.325-1.661) | <0.001 | 1.449(1.293-1.623) | <0.001 |
| Q3 (10.2[9.1-11.6]) | 871 | 1.694(1.523-1.885) | <0.001 | 1.612(1.445-1.797) | <0.001 | 1.476(1.320-1.650) | <0.001 |
| Q4 (13.9[>11.6]) | 920 | 1.866(1.679-2.074) | <0.001 | 1.907(1.712-2.125) | <0.001 | 1.816(1.624-2.030) | <0.001 |
| ***P* for *trend*** |  | <0.001 |  | <0.001 |  | <0.001 |  |
| **Female**, Total Bilirubin (μmol/L, Median [IQR]) | | |  |  |  |  |  |
| Q1 (5.315[≤6.4]) | 179 | 1(Reference) |  | 1(Reference) |  | 1(Reference) |  |
| Q2 (7.4[6.4-8.2]) | 236 | 1.378(1.135-1.673) | 0.001 | 1.237(0.917-1.651) | 0.148 | 1.208(0.896-1.629) | 0.214 |
| Q3 (9.2[8.2-10.3]) | 249 | 1.506(1.243-1.825) | <0.001 | 1.140(0.850-1.528) | 0.382 | 1.154(1.853-1.562) | 0.353 |
| Q4 (12.2[>10.3]) | 259 | 1.594(1.318-1.929) | <0.001 | 1.673(1.269-2.205) | <0.001 | 1.799(1.341-2.412) | <0.001 |
| ***P* for *trend*** |  | <0.001 |  | <0.001 |  | <0.001 |  |

HR and 95% CI for changes in IBIL for fundus arteriosclerosis incidence according to quartiles of TBIL in total population, male and female. ^*^: Model1, non-adjusted; ^†^: Model2, adjusted for age, BMI, FBG, smoke, drunk; ^‡^: Model3, adjusted for age, BMI, FBG, smoke, drunk, AST, ALT, γ-GT, TG, TP, Albumin, Globulin, CHOL, HDL, LDL, UA, BUN, hypertension status, diabetes status and fatty liver status.

HR: hazard ratio; CI: confidence interval; IQR: interquartile range.
